# Fault detection of taper roller bearings using tunable Q-factor wavelet transform and fault classification using long–short-term memory network

**DOI:** 10.1038/s41598-025-93514-3

**Published:** 2025-03-20

**Authors:** A. Anwarsha, Narendiranath Babu T

**Affiliations:** School of Mechanical Engineering, Vellore Institute of Technology, Vellore, Tamil Nadu 632 014 India

**Keywords:** Fault diagnosis, Taper roller bearing, Tunable q-factor wavelet transform, Deep learning, Long–short-term memory network, Aerospace engineering, Mechanical engineering

## Abstract

Taper roller bearing is a widely used moving component in heavy industrial machinery. Hence, early detection and repair of even minor faults in taper roller bearing is a fault diagnosis and prognosis strategy followed by modern industries. Although many methods for this exist today, the penetration of artificial intelligence and big data analysis into modern industries opens up the possibility of developing better fault diagnosis methods. Such a fault diagnosis and fault classification strategy is going to be discussed in this article. For that, a Tunable Q-factor Wavelet Transform (TQWT) is employed for signal processing, and a Long–Short-Term Memory (LSTM) network is employed for fault classification in this work. It is clear from the experimental findings that the TQWT and LSTM combination can very efficiently and reliably diagnose the faults present in the bearings, and it can classify the types of faults with one hundred percent accuracy. Also, the superiority of the method proposed in this article is confirmed by the fact that it is able to produce better results when compared with the other four combinations of Variational Mode Decomposition (VMD) and Convolutional Neural Network (CNN).

## Introduction

A rolling element bearing is a crucial element of every machine. A bearing’s function is to eliminate friction from the shaft’s rotation, making it very easy to rotate. Depending on the application requirements like load, speed, torque, etc., various bearing types are employed nowadays. Among these, taper roller bearings are a vital kind of bearing. They are commonly employed in heavy industrial machines because of their heavy load-carrying capacity, durability, and fatigue resistance. Taper roller bearings are susceptible to damage, much like any other movable components in a rotating machine. Machine breakdowns, catastrophic failure, etc. are all possibilities if it is damaged and neglected. So, the primary technique recommended by any contemporary industry is to continuously monitor the bearing’s health condition and rectify even the smallest damage.

This paragraph provides a brief explanation of how to keep monitoring a bearing’s health. Collecting the bearing data is the first stage. Data collection makes use of a range of sensors. These sensors include, for instance, accelerometers and acoustic emission sensors. Data processing is the following stage. Data processing is the process of using a computer to remove undesired components like noise from previously collected data so that it is ready for use. The third and last stage is fault identification and fault categorization. In this stage, the processed data is examined, and the health condition of the bearing is established from it. Many methods are used today for data processing and fault identification. In the early days, bearing health was determined by using any single method. However, the current trend is to combine two or more techniques to assess a bearing’s health. For example, use a method for data processing. From there we get the processed data. The denoised data thus obtained is then used to determine the bearing’s health with the help of some other classification algorithms. This kind of fault diagnosis is more transparent than any other conventional method and emphasizes high accuracy and reliability.

This paragraph summarizes some of the methods that have been applied to the subject of rolling element bearing defect diagnosis. The most common type of data utilized to find defects is vibration data. Data can be processed in several approaches, such as time-domain, frequency-domain, time–frequency domain, etc. The numerous forms of defect are also categorized using a variety of techniques. Machine learning and deep learning are two promising artificial intelligence approaches in this regard. A detailed explanation of each of the methods stated above can be found in references^1–10^.

The following paragraphs demonstrate how the tunable q-factor wavelet transform is employed to diagnose defects in rolling element bearings. Let’s start by listing the experiments that performed fault identification just utilizing the original TQWT approach. The fault in a rolling element bearing was discovered using the original TQWT approach by Kumar et al.^11^, and Gu et al.^12^. However, later researchers considered adding some other adjustments to the original TQWT to boost its effectiveness a little. The improved variants of TQWT used for rolling element bearing defect detection include double TQWT, adaptive TQWT, multi-q-factor multi-level TQWT, iterative TQWT, etc.^13–17^. All of the aforementioned techniques were excellent at fault detection, but some enhanced forms, like MQML-TQWT, required more computation. Similarly, we can comprehend why iterative TQWT is struggling to handle large amounts of data.

To get better results, however, some researchers integrated TQWT with additional signal processing methods. To detect faults in rolling element bearings, TQWT integrates signal processing methods such as neighboring coefficient denoising, ensemble empirical mode decomposition, overlapping group shrinkage, compressible sensing technique, matching pursuit algorithm, intrinsic characteristic scale decomposition, group sparsity total variance denoising method, orthogonal matching pursuit, multiscale statistical process control, etc.^18–24^. But these techniques have some drawbacks as well. Based on their dictionary density, algorithms like matching pursuit, for instance, work well. As a result, as dictionary density rises, computation costs will rise and dictionary density decreases, fault detection accuracy will decrease.

Further research articles revealed the usage of additional classification algorithms in addition to TQWT. Naive Bayes classifier, k-means clustering algorithm, and sparse representation classification algorithm are employed to identify the defects in rolling element bearings along with the TQWT^25,26^. Similar attempts have been made by other researchers to detect faults in rolling element bearings by combining TQWT with computational optimization techniques like particle swarm optimization. In Ma et al.^27^ performed the defect detection of bearings by integrating frequency slice wavelet transform and PSO with TQWT. Zhao et al.^28^ employed PSO in 2020 to identify the best q-factor for using TQWT in their trials to identify flaws in rolling element bearings. However, the computational complexity involved in employing these optimization techniques, together with the requirement for human knowledge and competence, are their limitations.

But later, some researchers began combining the TQWT method with artificial intelligence procedures like machine learning and deep learning, to identify rolling element bearing defects more precisely and reliably. Upadhyay and Kankar^29^ coupled TQWT with artificial neural networks, support vector machines, and decision trees to accurately classify the bearing failure. In their study, the TQWT method was used to break down the vibration signals collected from the experimental setup into different sub-bands. To accurately categorize the bearing flaws, certain statistical and fractal features were computed for every decomposed sub-band. ML techniques like ANN, SVM, and decision trees were then applied to the aforementioned features. To categorize various faults, present in the rolling element bearings, some researchers^30^ integrated TQWT with SVM, random-forest-tree classifier, and ANN in the same year. In their study, the permutation entropy features were computed for each decomposed time–frequency coefficient with the help of TQWT, and the fault features were classified as inner race fault, outer race fault, ball fault, and healthy bearing with the help of ANN, SVM, and RF.

In Zhang et al.^31^ proposed a wheelset bearing failure diagnosis using the TSCK-guided tunable Q-factor wavelet transform. This approach builds the Teager energy spectrum correlation kurtosis (TSCK) as the objective function, which is intentionally sensitive to periodic fault impulse components. In the same year, some researchers^32^ proposed the rolling bearing fault diagnosis method using Local Mean Decomposition (LMD) and Tunable-Q Wavelet Transform (TQWT) by considering the information about rolling bearing faults is relatively poor and there are interference signals. In Shi et al.^33^ published an article on wheel-set bearing defect diagnosis based on the bidirectional iterative merging multi-Q tunable-Q wavelet transform. The primary goal of this research is to identify recurrent transient impact signals and the optimal resonance band (ORB) in the axle box acceleration signals that have been collected. Recently, Rezazadeh et al.^34^ published a paper on optimizing a deep learning architecture to mitigate the effects of data distribution shifts in rotary machine failure detection.

Hou and Li^35^ integrated TQWT with convolution neural networks to categorize the rolling element bearing faults. In their study publication, Hou and Li discussed how to diagnose REB faults by using CNN and TQWT to reduce the impact of vibration signal noise and the time required for fault classification. The suggested work has been found to have a higher fault diagnosis accuracy and a greater capacity for generalization than the traditional approaches. Reference^36^ provides a thorough review of the use of TQWT in rolling element bearing defect diagnostics. In this review article, the TQWT application studies have been categorized into seven groups: These include TQWT fault diagnosis with other signal processing techniques, TQWT fault diagnosis with classification algorithms, TQWT fault diagnosis with computational optimization techniques, TQWT fault diagnosis with machine learning algorithms, and TQWT fault diagnosis with deep learning architectures.

*Motivation* This paragraph explains the motivation for choosing taper roller bearings for this study. First, ball bearings are the subject of the majority of research into the defect diagnostics of rolling element bearings. There haven’t been many studies on taper roller bearings. Similar to this, industrial machinery that needs to handle enormous weights frequently uses taper roller bearings. For these reasons, taper roller bearings were used for this research. It can be said that there are very few experiments on defect identification of REB by integrating TQWT with deep learning architectures. Therefore, there is still a lot of potential for future experiments that combine signal processing techniques like TQWT with artificial intelligence techniques like deep learning to conduct extremely accurate defect identification.

*Novelty* The vibration signal produced by the taper roller bearings is the source of the data used in this paper. It is clear from reading numerous studies in this area that the wavelet transform is most frequently employed for processing vibration signals. Although there are other wavelet transform variations, the tunable q-factor wavelet transform is the most sophisticated. The q-factor, often known as the quality factor, is a crucial wavelet transform parameter that can be easily modified, setting the TQWT apart from other common wavelet transform derivatives. That is, it is easy to find a sufficient q-factor and perfectly break down the signal to be evaluated by understanding its oscillatory nature. Likewise, for fault classification, the most advanced frameworks are deep learning algorithms. Long–short-term memory network, or LSTM, is employed in this case. Because it has the ability to store in long-term memory both current and past learning information relevant to a task. This LSTM property is highly helpful in the classification of faults. The objective of this research is to use a long–short-term memory network and tunable q-factor wavelet transform to develop a novel fault diagnostic and fault classification method for the taper rolling bearing.

The construction of this article is as follows: Section “Methodology” presents a brief explanation of the tunable q-factor wavelet transform, long–short-term-memory network, and the proposed work’s structure. Sections “Simulation analysis” and “Experimental Framework” respectively contain simulation analysis and the experimental framework. Section “Results and discussions” presents the results and the discussions and section “Feasibility for real-time applications” contains the feasibility of the method for real-time applications. Finally, section “Conclusions” provides the conclusions of this investigation.

## Methodology

TQWT and LSTM make up the two pillars of the suggested methodology. TQWT is used for signal processing, while an LSTM network is used for fault classification. By adjusting the transform parameters, TQWT provides significant flexibility for expressing signals with various temporal and spectral properties. Moreover, LSTM offers a higher classification accuracy to classify the various fault types seen in a rolling element bearing.

### Tunable Q-factor wavelet transform

Signal processing is substantially facilitated by the ability to tune the parameters in accordance with the oscillatory characteristics of the input signal. Selesnick’s TQWT^37^, a modified version of the wavelet transform, provides this capability. When a signal’s decomposition parameters, such as the q-factor, the decomposition level, and the redundancy factor, are set in accordance with the signal’s oscillatory nature, this is made possible. For instance, in this article, a taper roller bearing’s defect signal needs to be isolated. However, the bearing defect signal will not be the only one included in the raw vibration signal that we capture. It will also include the noise signal as well as the signal from other moving components of the same machine. Hence, it is quite challenging to isolate the bearing defect signal buried in other undesired signals. That is where TQWT can be of assistance. In contrast to the noisy signal, which is highly oscillatory, the bearing defect signal is less oscillatory in nature. So, we set the TQWT parameters suitable for less oscillatory signals and extract the fault signal from the jumbled raw vibration signal.

The following are the three parameters that the TQWT approach indicated in the preceding paragraph uses to help decompose signals.

*Q-Factor* The q-factor or the quality factor Q is the first parameter. It is by definition, the center frequency to bandwidth ratio, as given in Eq. 1. The q-factor expresses a signal’s oscillatory properties or the intensity of oscillation. The wavelets exhibit stronger oscillatory cycles for high q-factors, making them good candidates for the extraction of oscillatory components. The wavelets contain non-oscillatory elements for low q-factors, making them suitable for the extraction of transient components.

*Decomposition level* Decomposition level J is the second variable; it influences the wavelets’ frequency coverage. A higher J number causes the wavelets to encompass a greater frequency range. To include the lower frequency as much as possible, the value of J should be chosen as large as possible. However, the resulting coefficients could be challenging to comprehend with these many levels. It is often recommended to limit the number of levels to ensure that the level-J wavelet does not outlast the signal being analyzed in order to prevent this. This criterion allows for the greatest number of levels to be found as given in Eq. 2.

*Redundancy* The third one is redundancy R. In this context, it refers to the total oversampling rate of the transform, which is calculated by dividing the total number of wavelet coefficients by the signal length to which the TQWT is applied. A value of three or higher is suggested for the provided value of R, which must be greater than one. The expression of R is given in Eq. 3.

The Q-factor can be represented as,

1 $$Q = \frac{{\omega_{c} }}{BW}$$

where $\omega_{c}$ is the center frequency and BW is the bandwidth.

The highest possible number of stages of decomposition,

2 $$J_{max} = \left[ {\frac{log(\beta N/8)}{{{\text{log}}(1/\alpha )}}} \right]$$

where *N* denotes the size of the input signal. *Α* and *β* are the low-pass scaling factor and the high-pass scaling factor, respectively.

The redundancy is represented as,

3 $$R = \frac{\beta }{1 - \alpha }$$

The low-pass frequency response,

4 $$H_{l} \left( \omega \right) = \left\{ {\begin{array}{*{20}c} {1, \left| \omega \right| \le \left( {1 - \beta } \right)\pi } \\ {\theta \left( {\frac{{\omega + \left( {\beta - 1} \right)\pi }}{\alpha + \beta - 1}} \right), \left( {1 - \beta } \right)\pi < \left| \omega \right| < \alpha \pi } \\ {0, \alpha \pi \le \left| \omega \right| \le \pi } \\ \end{array} } \right.$$

The high-pass frequency response

5 $$H_{h} \left( \omega \right) = \left\{ {\begin{array}{*{20}c} {0, \left| \omega \right| \le \left( {1 - \beta } \right)\pi } \\ {\theta \left( {\frac{\alpha \pi - \omega }{{\alpha + \beta - 1}}} \right), \left( {1 - \beta } \right) < \left| \omega \right| < \alpha \pi } \\ {1, \alpha \pi \le \left| \omega \right| \le \pi } \\ \end{array} } \right.$$

where, $$0 < \alpha < 1, 0\left\langle {\beta \le 1, \alpha + \beta } \right\rangle 1$$.

And $$\theta \left( \omega \right) = 1/2\left( {1 + \cos \omega } \right)\sqrt {2 - \cos \omega } , {\text{for}} \left| \omega \right| \le \pi$$.

**Fig. 1:**
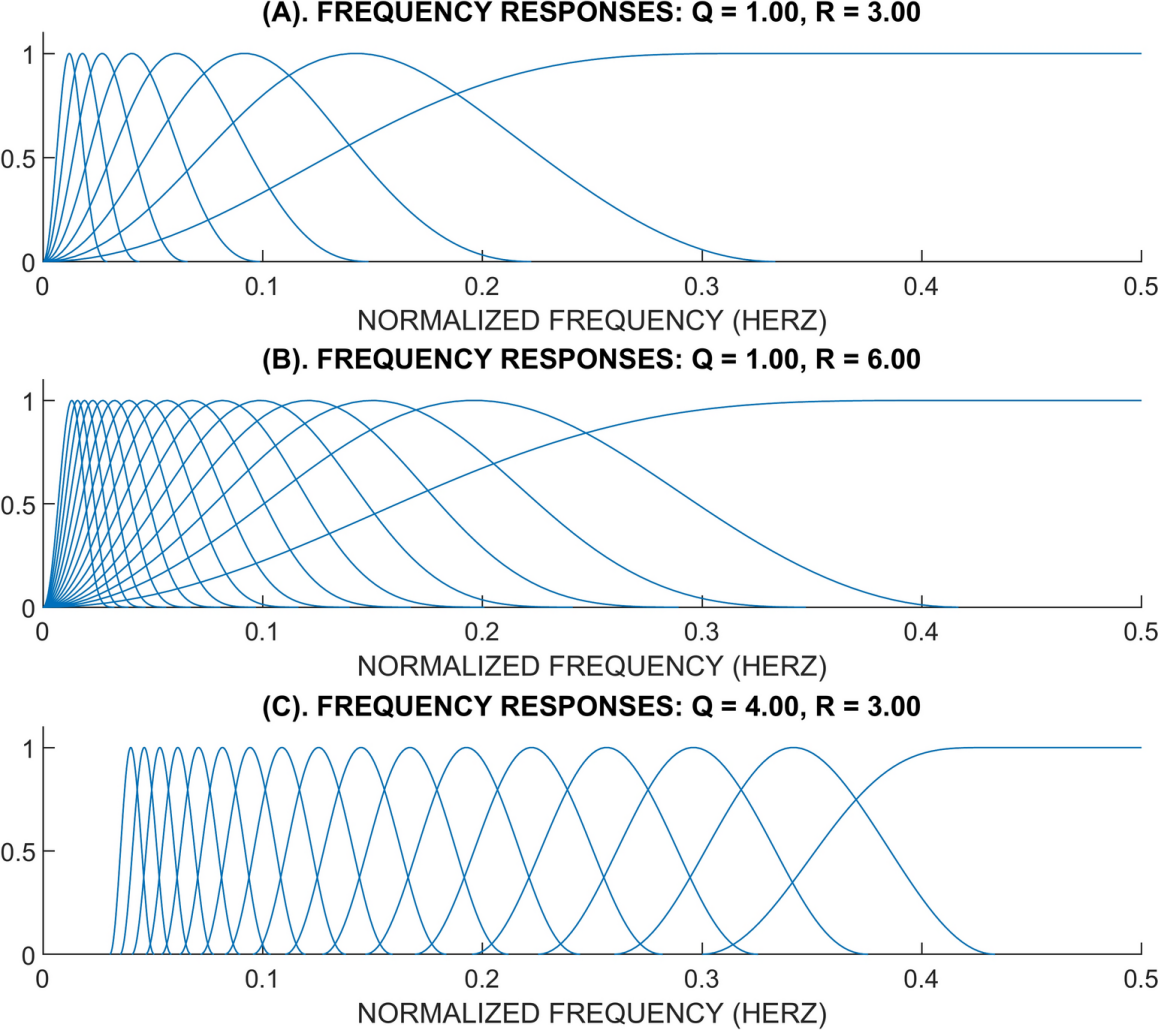
The frequency responses were acquired using TQWT.

It is essential to understand how the Q, R, and J components affect signal analysis. A frequency decomposition by TQWT example can be seen in Fig. 1. The overlap between adjacent frequency responses rises when R is raised while Q remains unchanged. The parameter R has no effect on the general form of the wavelet of frequency response. In order to cover a similar frequency range, level J should be increased with a larger R due to the higher overlap. When comparing Fig. 1A and B, it can be seen that when the R is bigger, adjacent bands overlap more. It is evident from looking at Fig. 1C that as Q rises, the wavelets become increasingly oscillatory. If Q is increased from 1 to 4, the frequency responses become narrower, hence more stages are needed to cover the same frequency range.

**Fig. 2:**
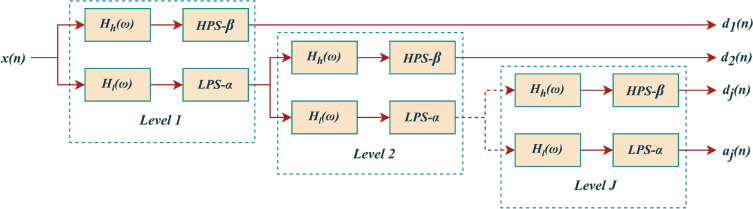
Analysis filter bank of TQWT.

**Fig. 3:**
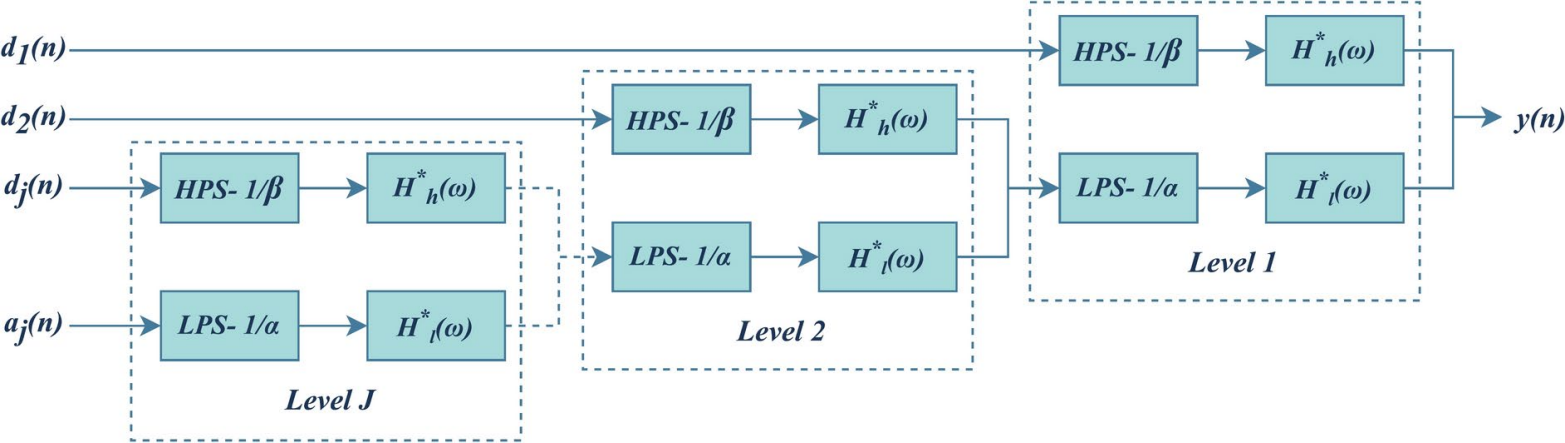
Synthesis filter bank of TQWT.

By iteratively applying the two-channel filter bank (analysis filter bank and synthesis filter bank as shown in Figs. 2 and 3) on its low-pass channel, the tunable q-factor wavelet transform is created. These filter banks’ perfect reconstruction characteristic is inherited via the wavelet transform. We must examine the iteration of various filters and scaling in order to determine the frequency decomposition that the wavelet transform offers. The corresponding responses of frequency for the low-pass and high-pass filters used in the two-channel filters utilized for each level are defined by Eqs. 4 and 5.

### Long- short-term memory network

**Fig. 4:**
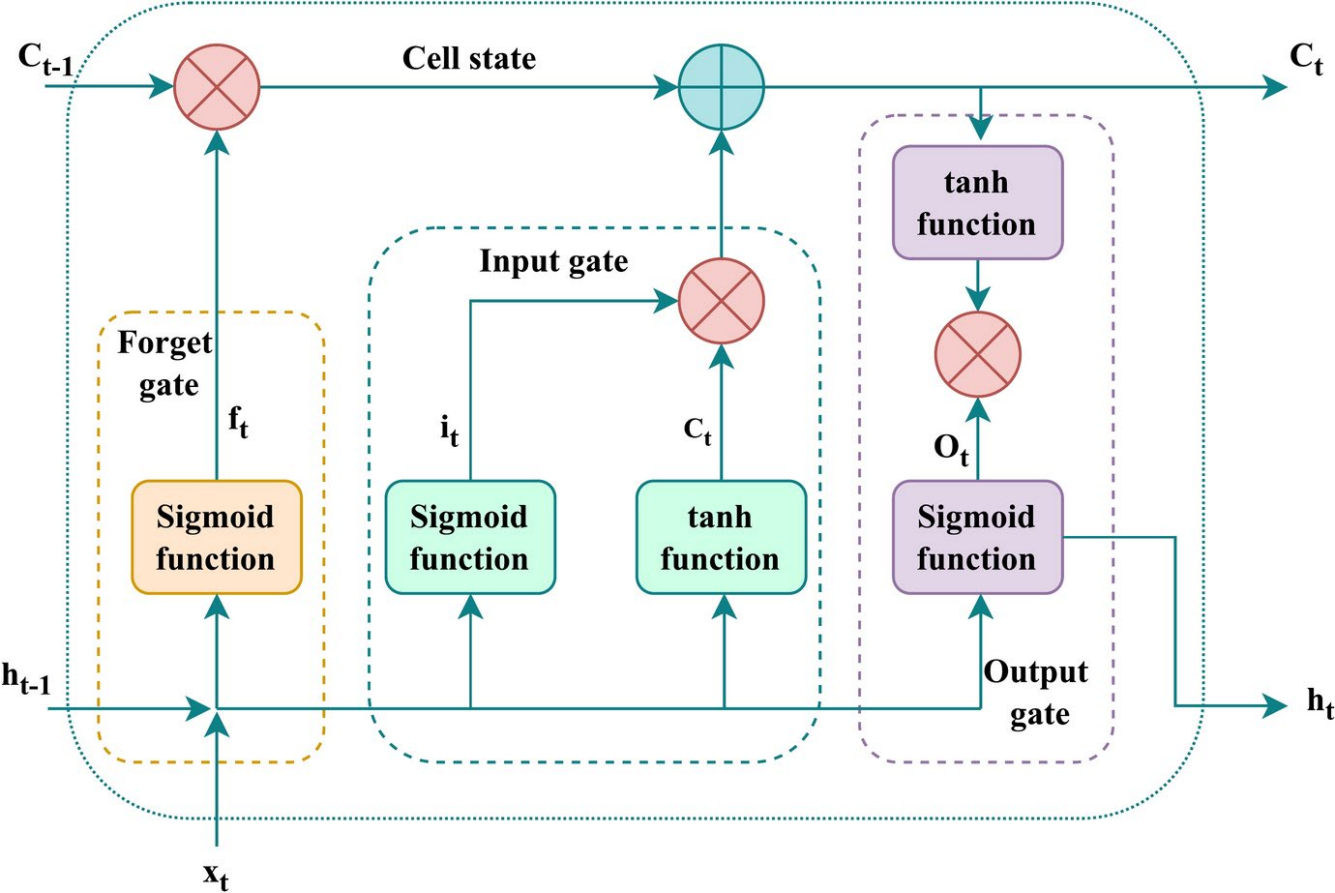
Structure of an LSTM network.

In contrast to standard Recurrent Neural Network (RNN) designs, the long–short-term memory network can solve the issue of fading gradients. The LSTM framework stands out from other frameworks because of its ability to save both recent and previous learning data for a task in long-term memory^38,39^. As shown in Fig. 4, an LSTM network has four gates: an input gate, a forget gate, an update gate, and an output gate. Let’s start by discussing the forget gate. The facts that should be remembered and ignored are determined by this gate. The sigmoid operator evaluates whether the past output data is necessary using the present input *x*_*t*_ and the prior hidden stage *h*_*t−*1_. Later, the value of *f*_*t*_ will be used. The forget gate at time *t* can be expressed using Eq. 6.

The cell state is updated by the input gate, which is the following component. The prior hidden state, *h*_*t-1*_, and the present input state, *x*_*t*_, are given to the second sigmoid function. From 0 to 1, the values are modified. The first is for critical information, and the second is for less crucial data. The identical information from the hidden stage and present state will then be passed on using the *tanh* operator. To control the framework, the *tanh* function creates a vector *c*_*t*_ with all feasible values between -1 and 1. The output values produced by the activation functions are prepared for point-by-point multiplication. The input gate can be expressed using Eq. 7.

Next is the update gate, also referred to as the cell state. The network has received enough data from the input gate and forget gate. It is therefore necessary to decide and reserve the current status data in the cell-state. The value of *f*_*t*_ magnifies the previous cell-state *C*_*t−*1_. The framework updates the cell state to a new cell state, *C*_*t*_, after summarizing the result value of the input vector.

Last but not least, the output gate regulates the price of the subsequent hidden step. Information from earlier inputs is saved during this stage. The values for the current stage and previous hidden stage are fed to the third sigmoid-function first. The previous cell state is then employed to create an updated cell-state using the *tanh* operator. Depending on the outcome, the network decides which information the hidden state should reveal. This hidden state serves as the foundation for prediction. The most recent cell state and hidden state are then advanced to the following stage. The expression for the update gate and the output gates are given from Eqs. 8–11.

In the aforementioned formulas *i*_*t*_, *f*_*t*_, *C*_*t*_, and *O*_*t*_ stand for input, forget, cell, and output gates, respectively. The current input and LSTM output are denoted by *x*_*t*_ and *h*_*t*_, respectively. The sigmoid operator and the outcome of the tanh operator are *σ* and *c*_*t*_. The weight matrices for the forget, input, cell, and output gates are *w*_*f*_, *w*_*i*_, *w*_*c*_, and *w*_*o*_, respectively. The previous step’s hidden and cell states are *h*_*t-1*_ and *C*_*t-1*_. The connection biases of forget, input, cell, and output gates are *b*_*f*_, *b*_*i*_, *b*_*c*_, and *b*_*o*_.

6 $$f_{t} = \sigma \left( {w_{f} .\left[ {h_{t - 1} ,x_{t} } \right] + b_{f} } \right)$$

7 $$i_{t} = \sigma \left( {w_{i} \left[ {h_{t - 1} ,x_{t} } \right] + b_{i} } \right)$$

8 $$c_{t} = {\text{tanh}}\left( {w_{c} \left[ {h_{t - 1} ,x_{t} } \right] + b_{c} } \right)$$

9 $$C_{t} = f_{t} *C_{t - 1} + i_{t} *c_{t}$$

10 $$O_{t} = \sigma \left( {w_{o} \left[ {h_{t - 1} ,x_{t} } \right] + b_{o} } \right)$$

11 $$h_{t} = O_{t} *{\text{tanh}}\left( {C_{t} } \right)$$

### The proposed method

This part describes the application of the unique proposed methodology to the structure of fault diagnosis and fault classification of a taper roller bearing. The entire procedure is divided into three stages: signal acquisition comes first, followed by signal processing and fault diagnosis, and finally fault classification.

Discussing signal acquisition will be helpful. In a defect diagnostic process, getting a good signal to examine without losing any vital data is important. The signal that was collected must be stored on a hard drive safely as well. The phase of signal acquisition is finished when these two things come together. This is achieved using accelerometers and a DAQ system.

**Fig. 5:**
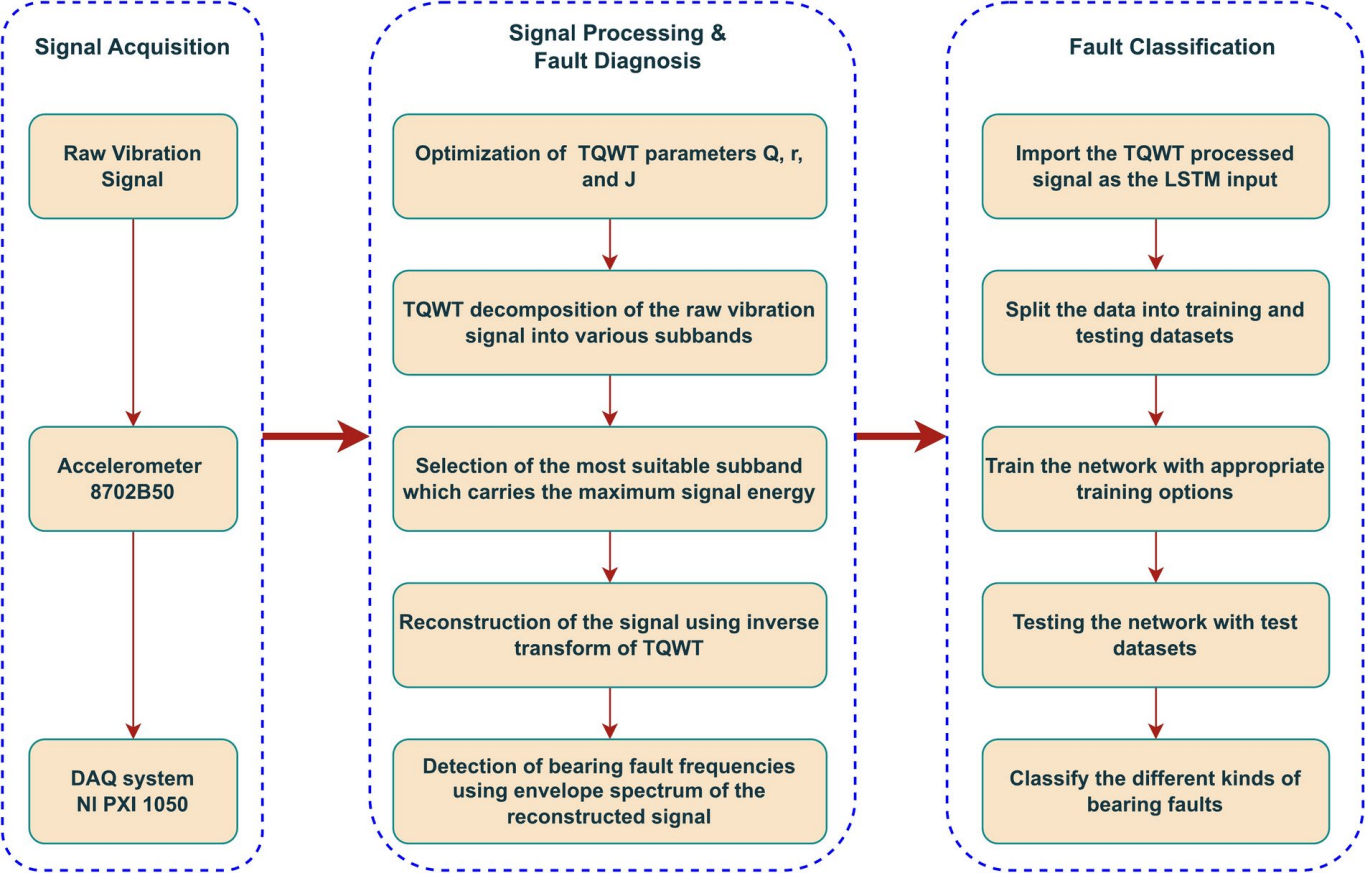
The framework of the proposed method.

The signal processing and fault diagnosis come second. A flawless diagnosis procedure requires a signal that is treated more effectively. It entails noise reduction, fault feature extraction, enrichment of fault features, fault characteristic frequency identification, separation of the defect frequency from other system components’ rotating frequencies, and so forth. Signal processing should also be dependable, time-efficient, and simple to execute. The tunable q-factor wavelet transform method is used to accomplish this task. Figure 5 outlines its precise steps. The TQWT parameters Q, R, and J are first optimized. High-energy signals are utilized to optimize the q-factor since they contain more information. A low q-factor is preferred since the fault signals are less oscillatory in nature. The q-factor is therefore selected from 1 to 3. Each type of defect signal is processed by first determining the signal energy using q-factor values between 1 and 3, and then optimizing the q-factor that produces the highest signal energy. A step size of 0.5 is used for optimization. Once the q-factor has been optimized, choose the proper R and J values. In the present research, we set R = 3 for computational efficiency. Equation 2 is used in this paper to limit the greatest level of breakdown. For each type of fault, this optimization procedure is repeated. The raw vibration signal that was previously recorded and stored is then divided into several subbands using the optimum parameters of TQWT. Since the greatest energy subband reflects the fault characteristic frequency, it is selected as the most appropriate subband among those thus produced. Utilize this subband to perform the inverse TQWT signal reconstruction. The fault frequency is then separated from the reconstructed signal’s envelope spectrum.

The classification of faults comes in third. Inner ring failure, outer ring failure, roller failure, and cage failure are the four types of failures that frequently occur in a taper roller bearing. These failures should be precisely categorized by a perfect classification algorithm. This is accomplished using the LSTM network. The LSTM approach uses the TQWT-processed signal as its input data. The data must be prepared to work with the LSTM network. The full collection of data is split into training and testing datasets for that. The network is then trained using the proper training options, evaluated using the test dataset, and the type of bearing failure is finally determined.

## Simulation analysis

**Fig. 6:**
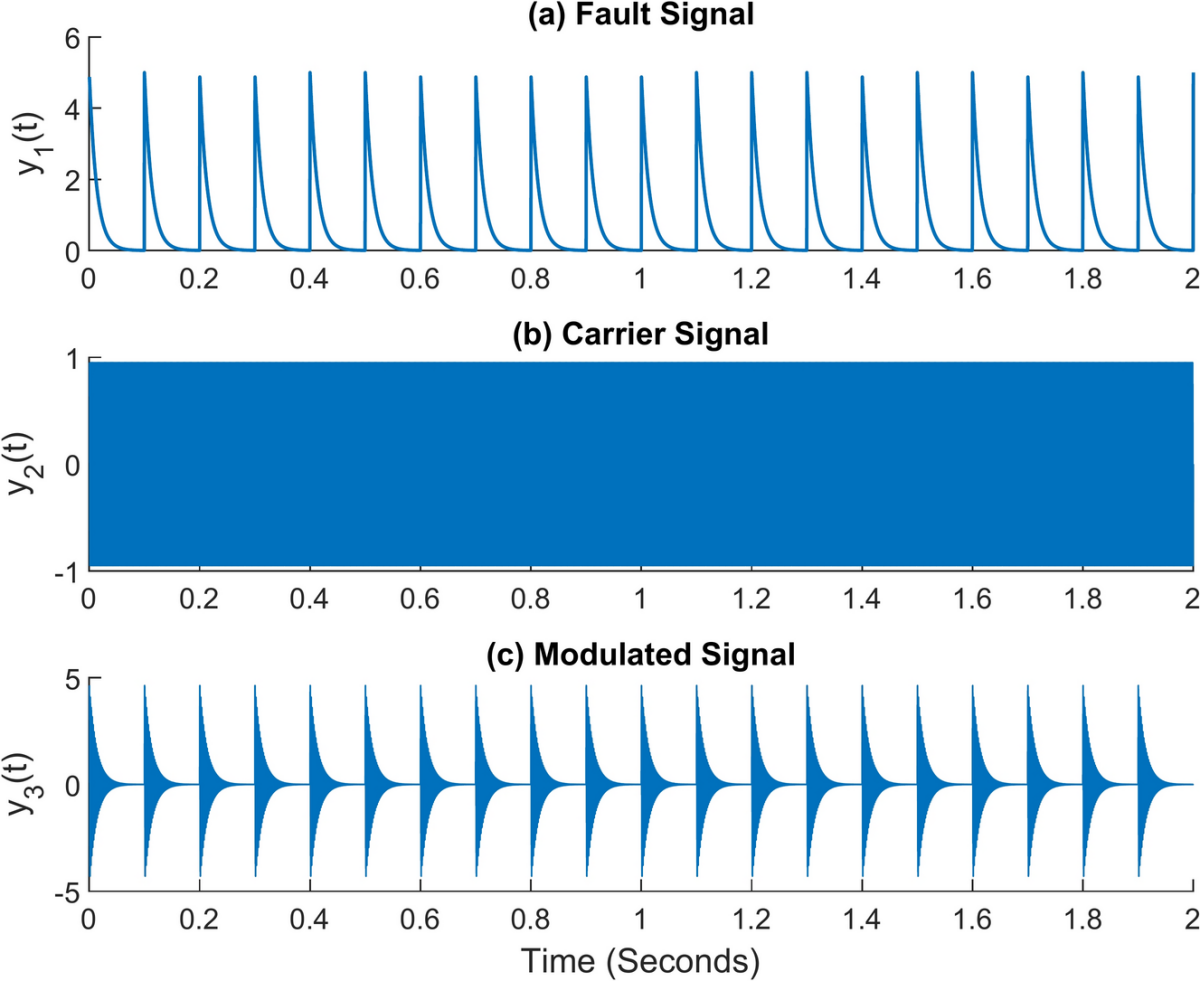
Simulated signals (**a**) Fault signal (**b**) Carrier signal (**c**) Modulated signal.

The simulated signal used in this paper closely approaches the actual signal. To achieve that, MATLAB is used to produce various signal types and combine them into a single signal. First, a fault signal with a frequency of 10 Hz must be produced, and the expression for doing so is provided in Eq. 12. Thusly produced, the fault signal is modulated on a different carrier signal. Equation 13 contains the expression for the modulating carrier signal. Such a carrier signal has a frequency that is substantially greater than the frequency of the defect signal. It is 600 Hz here. This is consistent with the bearing’s resonance. In Eq. 14, the expression for the modulated fault signal is thus provided. Figure 6a–c, respectively, show the time domain graphs of the fault signal, carrier signal or resonance signal, and modulated signal.

**Fig. 7:**
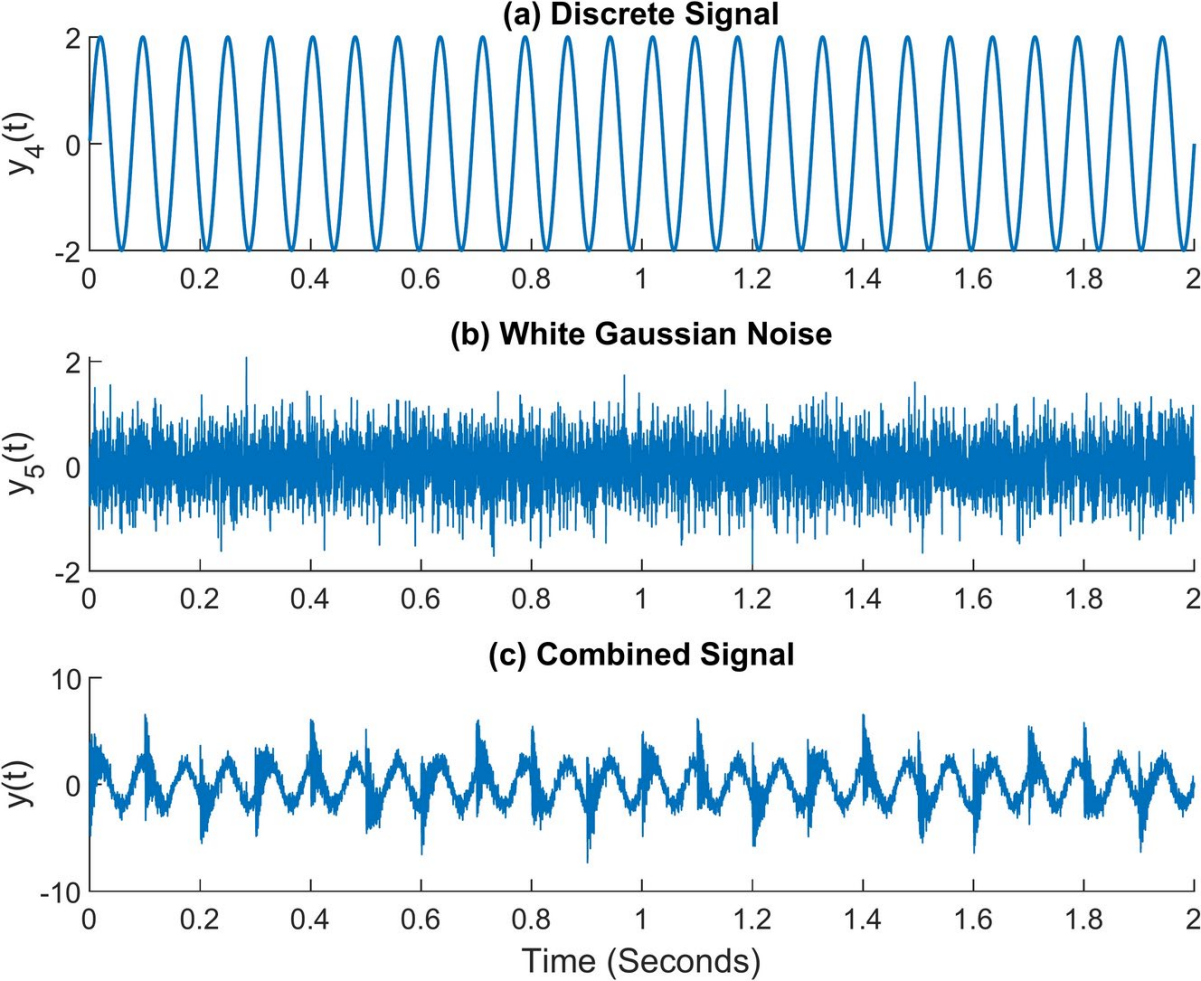
Simulated signals (**a**) Discrete signal (**b**) White Gaussian noise (**c**) Combined signal.

**Fig. 8:**
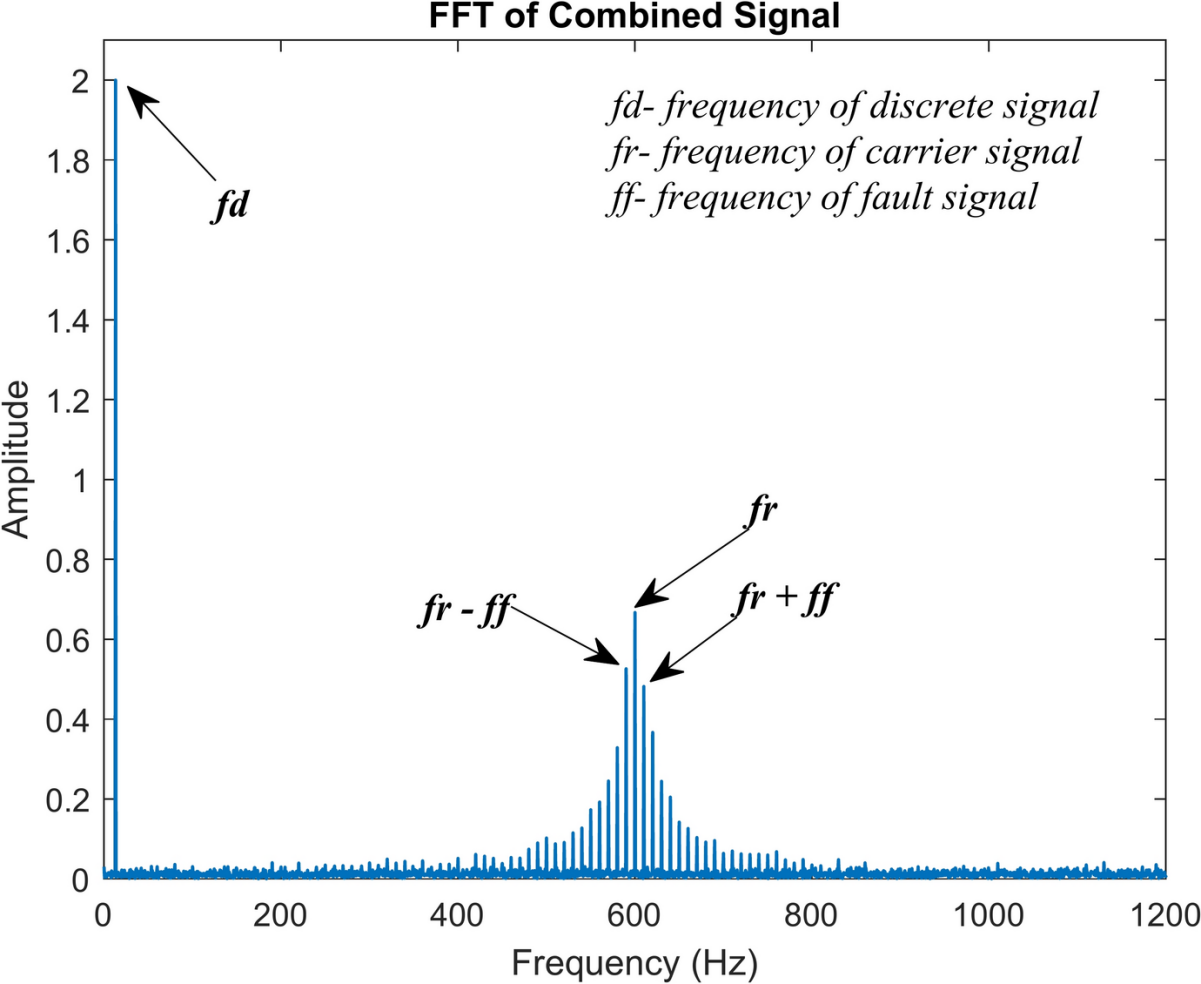
FFT of the combined signal.

In addition to this, real-time working conditions will include the presence of numerous other indications. For instance, when a piece of machinery operates, vibration signals are also produced by the gears, shafts, and other moving parts of the machine. For that, the discrete signal expression in Eq. 15 might be taken into consideration. It takes in an amplitude of 2 and a frequency of 13 Hz. The ambient noise must also be considered. Equation 16 provides the equation for white Gaussian noise. All of the signals are then added together to form a single composite signal. Equation 17 contains the expression for the combined signal. Figure 7a–c, respectively, show the time domain graphs of the discrete signal, white Gaussian noise, and combined signal. Figure 8 shows the combined signal’s fast Fourier transform (FFT). The carrier signal and its sidebands are responsible for the high-frequency region’s amplitudes. The discrete signal and the modulated signal have amplitudes of 2 and 0.6, respectively. It denotes that the carrier signal and other discrete signals have buried the actual fault signal^40^.

12 $$y_{1} = A.e^{{ - \left( {2\pi .a.f_{r} .t} \right)}}$$

13 $$y_{2} = \sin \left( {2\pi .f_{r} .t} \right)$$

14 $$y_{3} = y_{1} .y_{2}$$

15 $$y_{4} = 2\sin \left( {2\pi .f_{d} .t} \right)$$

16 $$y_{5} \approx N\left( {0,\sigma^{2} } \right)$$

17 $$y = y_{3} + y_{4} + y_{5}$$

In the equations, *A* denotes the signal’s maximum amplitude. It is assumed to be 5 in this example. The damping ratio, *a*, is assumed to be 0.02. The carrier signal’s frequency, denoted by the letter *f*_*r*_, is 600 Hz in this instance. The discrete signal’s frequency is denoted as *f*_*d*_, it is assumed to be 13 Hz, and *t* represents time in seconds. The variance of the white Gaussian noise signal is ${\sigma }^{2}$; in this instance, it is taken to be 0.5.

**Fig. 9:**
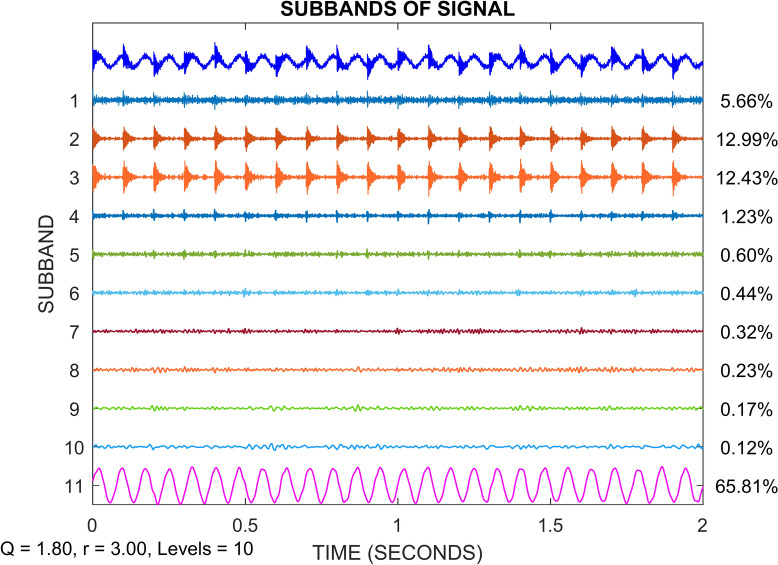
TQWT decomposition of the simulated signal into J + 1 subbands.

The combined simulated signal mentioned in the previous paragraph is put through the TQWT decomposition as the next step in this section. The TQWT settings must be selected for that. We take Q = 1.8, R = 3, and J = 10 at this moment. Figure 9 shows the TQWT decomposition result. Where J + 1 subbands are created by decomposing the combined simulated signal. In which the last subband (the eleventh subband) corresponds to the approximation subband and the previous ten subbands belong to the detail subbands. The distribution of the signal energy is also depicted in Fig. 9. The maximum signal energy among the detail subbands is carried by subbands 2 and 3, which are 12.99% and 12.43% of the overall energy, respectively. Only a small quantity of energy is carried by the remaining detail subbands. The next step is to reconstruct the signal using the inverse TQWT method. For reconstruction, the two detail subbands with the highest signal energy are used (subbands 2 &3).

**Fig. 10:**
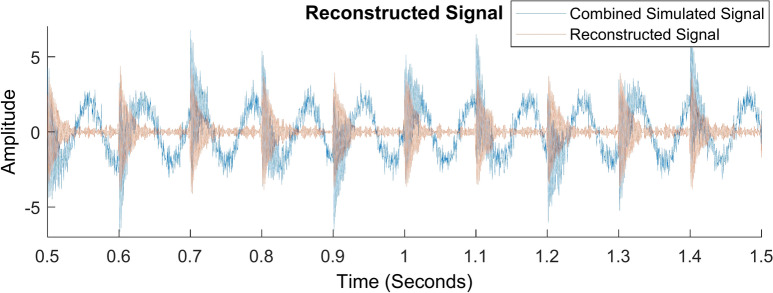
Reconstructed signal using inverse TQWT.

**Fig. 11:**
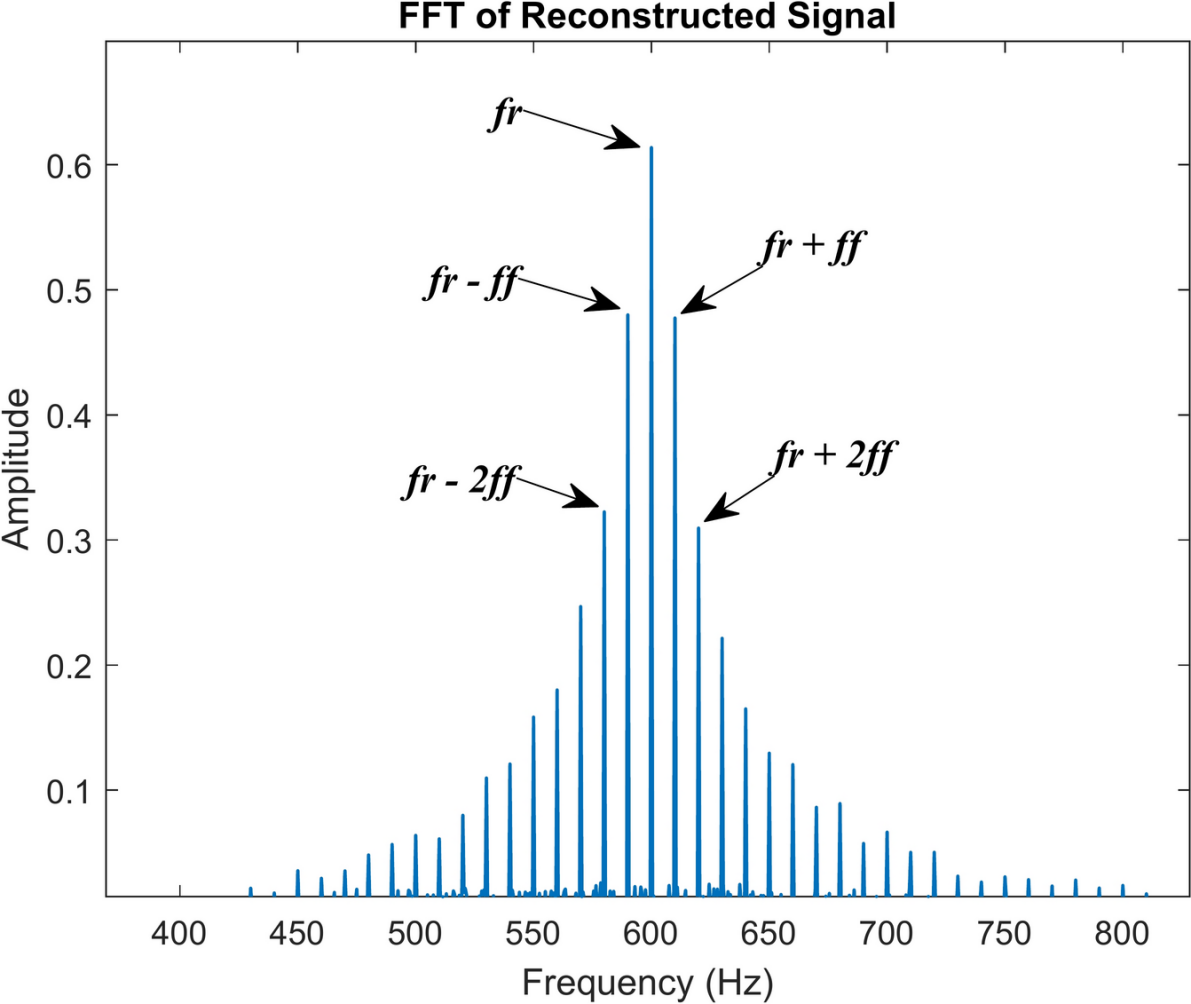
FFT of the reconstructed signal.

The reconstruction of signals using the inverse TQWT approach is shown in Fig. 10. It compares the combined simulated signal and the signal that was reconstructed after TQWT was used. As can be observed, the modulated fault signal provided in Fig. 6c and the reconstructed signal following the TQWT operation are comparable. The fast Fourier transform of the reconstructed signal is given in Fig. 11. Without any other disturbances brought about by discrete signals or noise, the modulated fault signal and its harmonics are clearly visible. This example demonstrates how the TQWT approach may accurately determine the fault characteristic frequency of a signal.

## Experimental framework

**Fig. 12:**
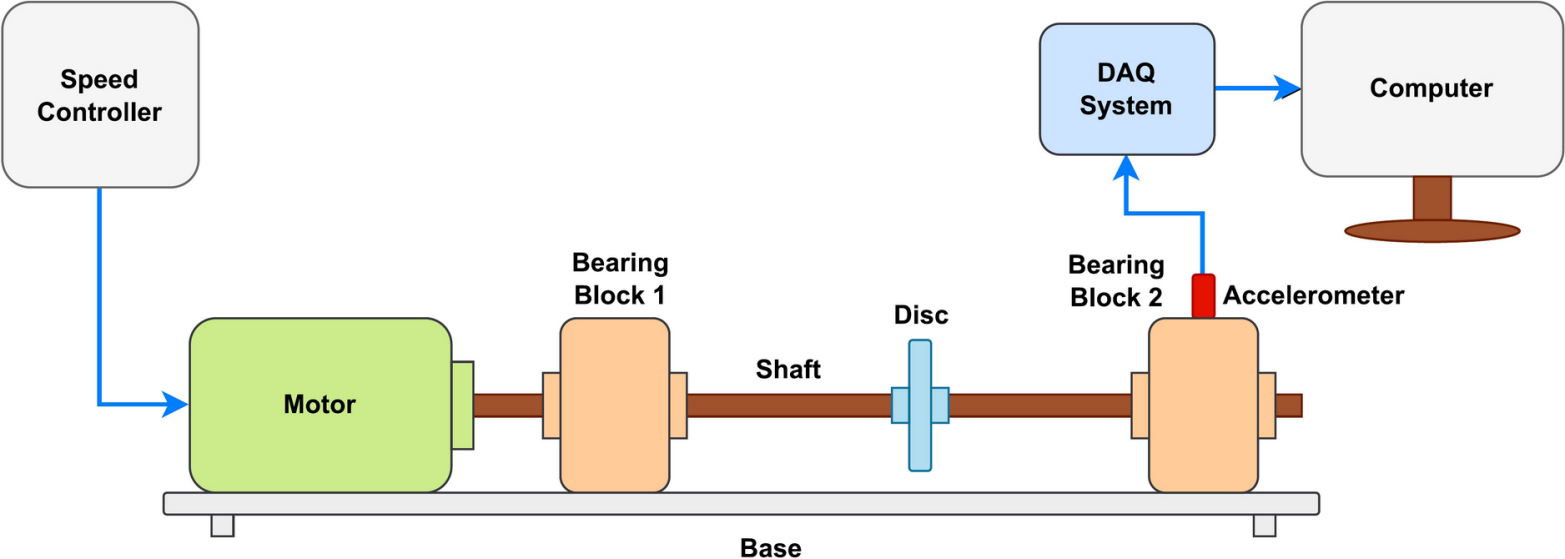
Schematic representation of the experimental setup.

**Fig. 13:**
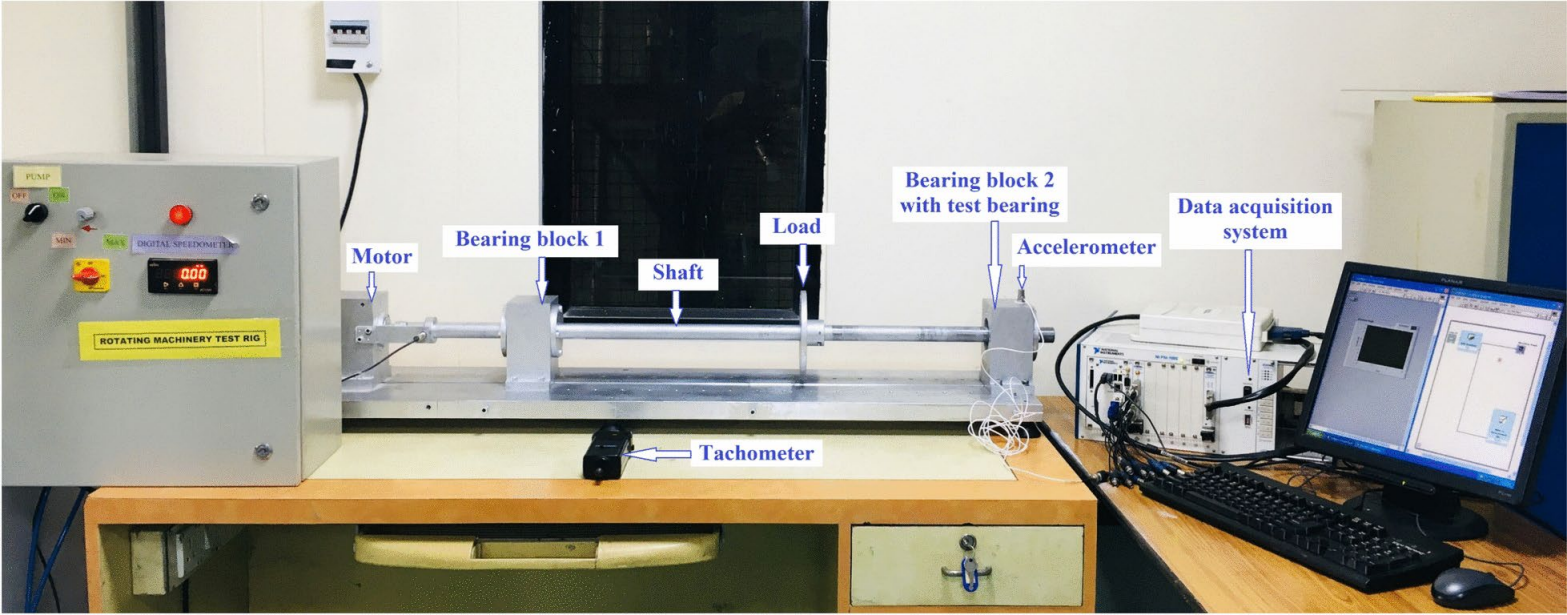
The experimental setup.

Figures 12 and 13 depict the experimental configuration. This includes a 30 mm diameter and 120 mm long shaft, a water-cooled, three-phase induction motor, a digital tachometer, two bearing blocks with flanges, and the test bearing housed inside bearing block 2. An accelerometer of type 8702B50 with a frequency response of 0.5–10,000 Hz, a sensitivity of 100 mV/g, and a range of ± 50 g is used to collect the vibration data. The accelerometer is mounted on the top surface of bearing block 2 in the 12 o’clock position to the test bearing. The accelerometer is connected to the 13th channel of a 14-channel data acquisition system, provided by National Instrument, model number NI PXI 1050.

**Fig. 14:**
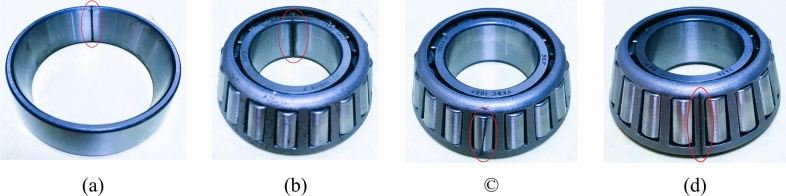
Test bearing defects (**a**) Outer ring (**b**) Inner ring (**c**) Roller (**d**) Cage.

The data were collected under a variety of circumstances, including variable shaft speeds, variable loads, and fault varieties. Five taper roller bearings are tested in this experiment. A normal (healthy bearing) is number one, followed by outer ring fault, inner ring fault, roller fault, and finally cage fault as shown in Fig. 14. The test bearing’s number is SKF 32,206, and its specifications are given in Table 1. The sampling frequency rate of the experiment is set as 20 kHz. All the faults were artificially created with the wire-cut electric discharge machining operation, and the fault’s length extends from one side to the other, with a width and depth of 0.25 mm each. There are further techniques, such as engraving and laser machining, that can create artificial faults on the races of the roller bearings. However, given the challenges associated with preserving geometric consistency, EDM appears to be more promising in terms of creating accurate artificial bearing faults in a practical and cost-effective manner. Regardless of hardness, its non-contact machining characteristics enable it to create artificial defects that precisely resemble naturally occurring faults. Additionally, EDM is quite capable of controlling variable defect size and appropriateness in defect shape^41^.

**Table 1:** Bearing specifications.

Specification	Value	Specification	Value
Bore diameter	30 mm	Width, outer ring	17 mm
Outside diameter	62 mm	Width, total	21.25 mm
Width, inner ring	20 mm	Contact angle	14.036°

The experiment began with a healthy bearing under different load conditions at various shaft speeds between 300 to 3400 rpm. Then repeat the experiment with several faulty bearings attached and collect the results. The vibration data is stored in the computer with the help of the installed version of National Instrument’s LabView software. The experiments were run on an Intel(R) Core(TM) i7-8565U CPU @ 1.80 GHz, and an installed RAM of 8.00 GB. The stored data is then transferred to the MATLAB environment for further signal processing. Section “Results and discussions” contains the testing results. All of the experimental results have been verified using the theoretical formulas for fault frequencies given in Table 2 and the manufacturer’s website for SKF bearings, referenced as^42^. In the formulas, *n* represents the number of rollers, here it is 17, *f*_*s*_ represents shaft rotation speed, here it is 300 to 3400 rpm, *d* represents the roller diameter, here it is 7.8 mm, *D* represents the pitch diameter, here it is 46 mm and *α* represents the contact angle, here it is 14.036^0^.

**Table 2:** Frequencies of defects across different categories.

Defect type	Defect representation	Defect frequency
Outer race fault	Ball pass frequency outer race, BPFO	$\frac{n}{2}f_{s} \left( {1 - \frac{d}{D}cos \propto } \right)$
Inner race fault	Ball pass frequency inner race, BPFI	$\frac{n}{2}f_{s} \left( {1 + \frac{d}{D}cos \propto } \right)$
Cage fault	Fundamental train frequency, FTF	$\frac{{f_{s} }}{2}\left( {1 - \frac{d}{D}cos \propto } \right)$
Roller fault	Ball or roller spin frequency, BSF	$\frac{{D.f_{s} }}{2d}\left[ {1 - \left( {\frac{d}{D}cos \propto } \right)^{2} } \right]$

## Results and discussions

This section provides the experimental findings and the discussions of the proposed methodology for fault identification and the fault classification of a taper roller bearing. The entire procedure is divided into two stages, the first stage is the signal processing and fault diagnosis by TQWT, and the second stage is the fault classification by the LSTM network.

### Signal processing and fault diagnosis by TQWT

This is the first stage of the proposed method; this stage explains how to use the TQWT method to determine the characteristic frequency of the various faults present in the test bearing. Signal processing and fault diagnosis by TQWT are carried out using each set of vibration data with a sampling frequency of 20 kHz and a data length of 32,768.

The q-factor, the number of decomposition levels J, and the redundancy R determine the decomposition performance of the TQWT technique. The oscillation of the wavelet base, which is reflected in the q-factor, is important for its decomposition performance. So, the q-factor needs to be optimized first. Since a high-energy signal has more information, the signal energy is used to optimize the q-factor. Since the fault signals are less oscillatory in character, a low q-factor is preferable. Therefore, the q-factor is chosen between 1 and 3. As we process each kind of fault signal, use the q-factor values ranging from 1 to 3 to assess the signal energy and then optimize the q-factor that yields the maximum signal energy. The optimization step size is selected to be 0.5. Select the appropriate R value and J value for the q-factor after optimizing it. For computational efficiency, we set R = 3 in this paper. The maximum level of decomposition is limited in this paper using Eq. 2. This optimization process is repeated for every kind of fault.

**Fig. 15:**
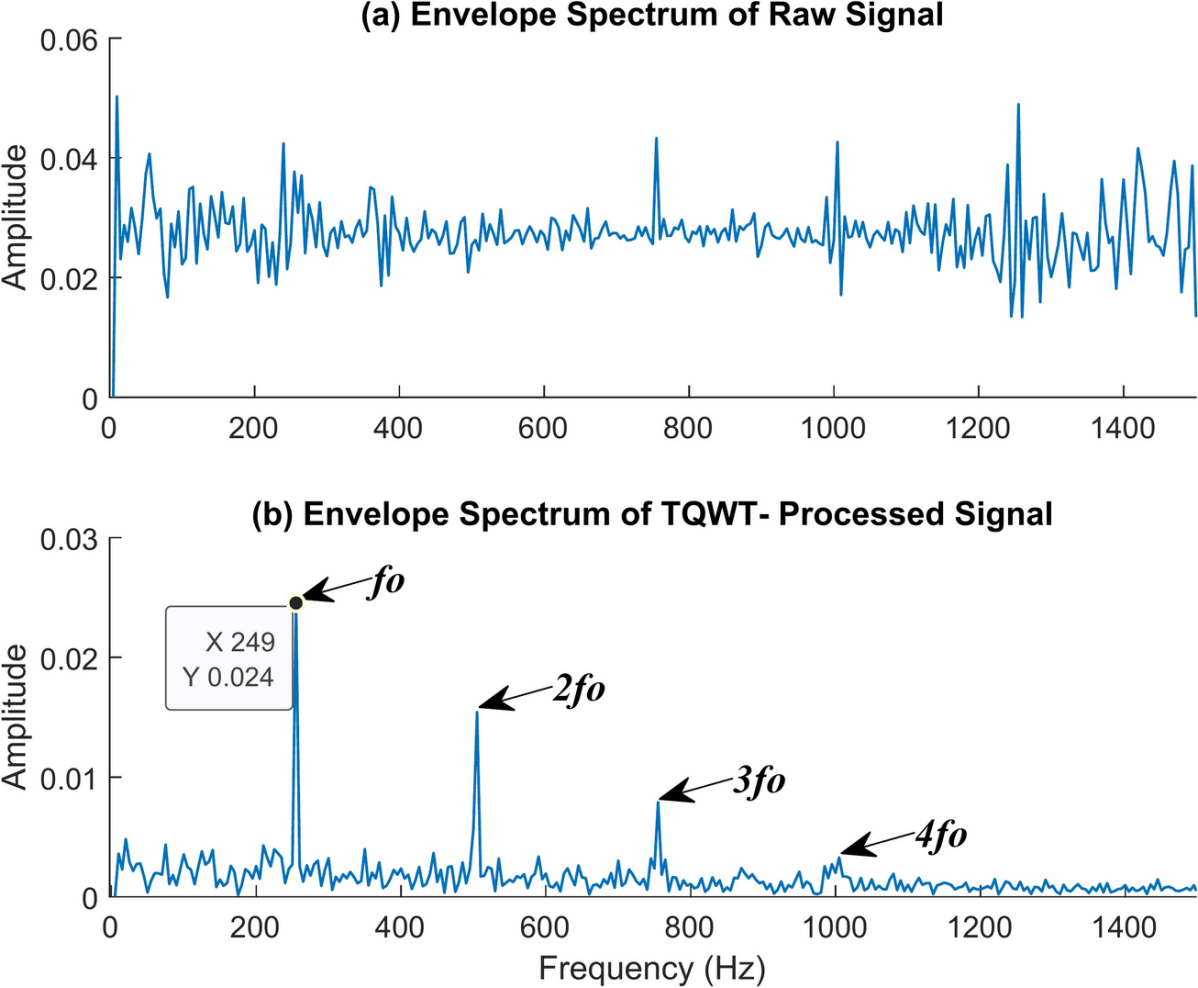
The envelope spectrum (ES) of the outer race fault rotates at 2100 rpm (**a**) ES of the raw signal (**b**) ES of the TQWT-processed signal (Q = 1.5, J = 10).

**Fig. 16:**
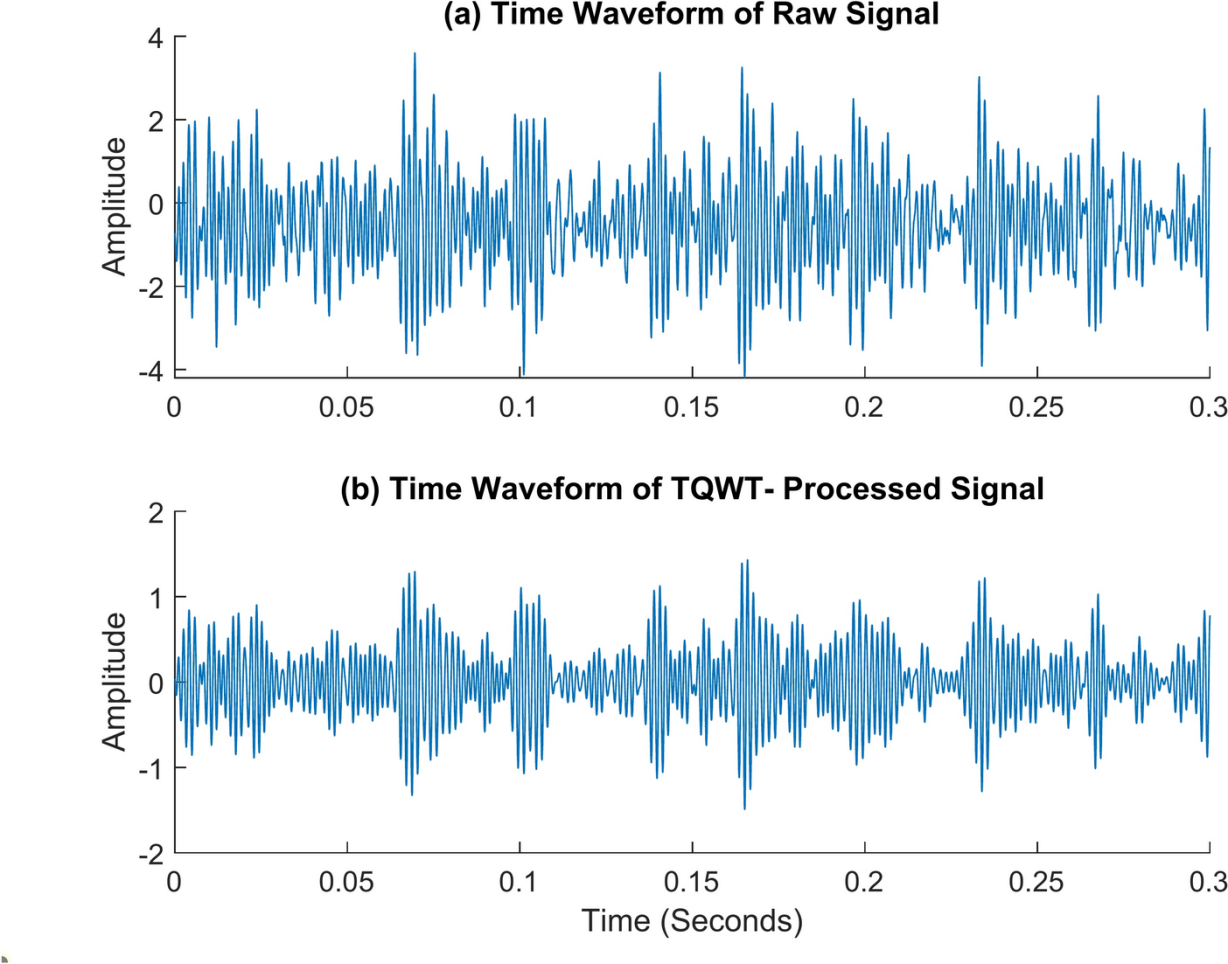
The time waveform (TW) of the outer race fault rotates at 2100 rpm. (**a**) TW of the raw signal (**b**) TW of the TQWT- processed signal (Q = 1.5, J = 10).

Begin with the outer race fault. For it, Q is optimized as 1.5, and the corresponding R value and J value are chosen as 3 and 10 respectively. The raw signal is decomposed into eleven subbands, out of which ten are detailed subbands, and one approximation subband. Then choose the most suitable detailed subband that contains the greatest energy and determine the fault frequency by using the envelope spectrum. Checking Figs. 15 and 16 can help you understand the extraction quality of the TQWT for an outer race fault at a shaft rotation speed of 2100 rpm, and at no load condition. Let’s start by examining Fig. 15. It contrasts the envelope spectrum (ES) of the TQWT-processed signal with the raw signal. The ES of the raw signal given in Fig. 15a does not provide us with any useful information. This indicates that the defect frequency is mixed in with the other signal frequencies. Yet, the outer ring fault frequency and associated harmonics can be plainly observed in the envelope spectrum of the TQWT-processed signal shown in Fig. 15b. The outer race fault frequency of 249 Hz is found, and this value is extremely close to the theoretical value of 248.5 Hz determined using the formula given in Table 2. In the figure, *f*_*o*_ represents the fault frequency of the outer race, *2f*_*o*_, *3f*_*o*_, and *4f*_*o*_ are its harmonics. In other words, when the TQWT is used, the noise and undesired signals are eliminated, leaving only the fault signal to be identified. The time waveforms of the raw and TQWT-processed signals are observed in Fig. 16.

**Fig. 17:**
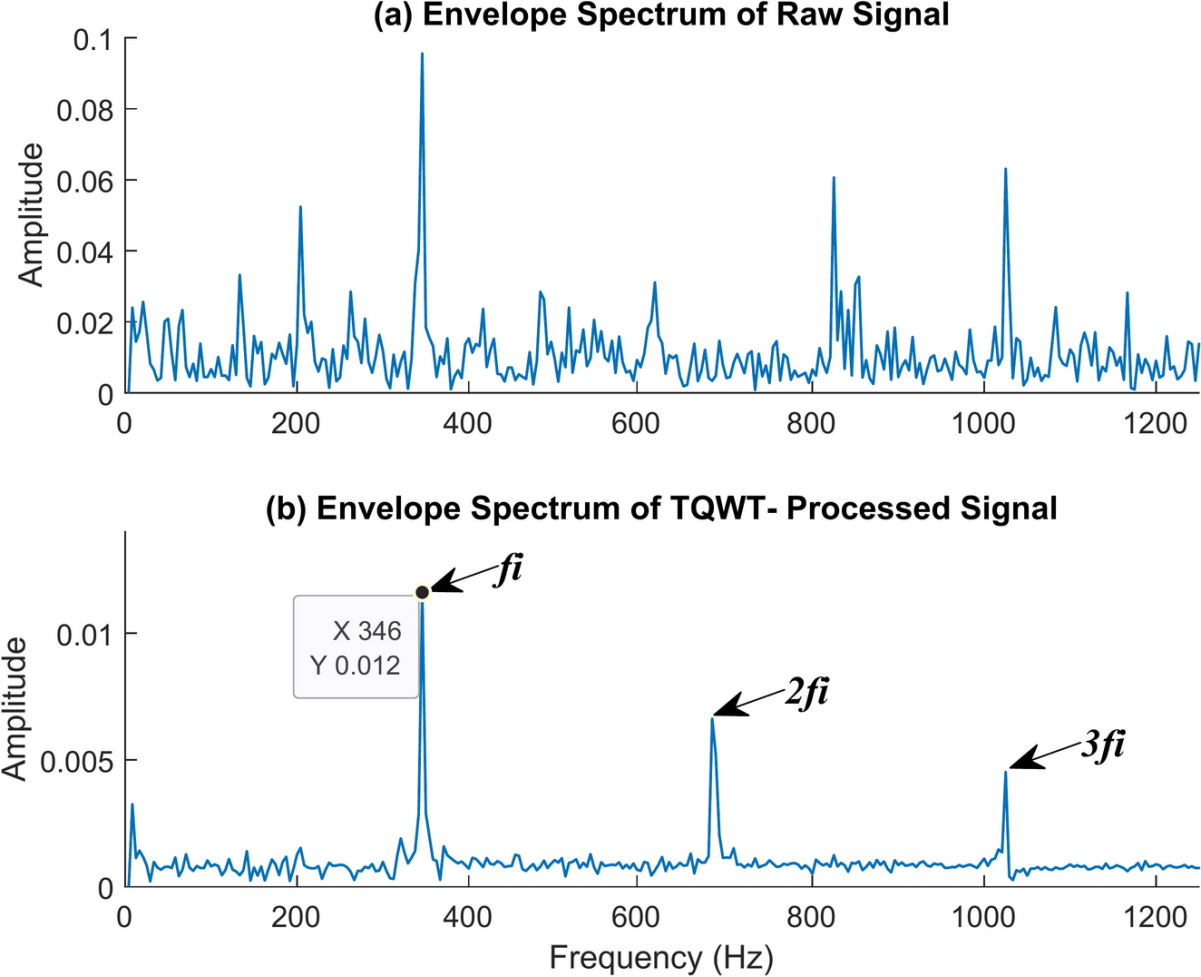
The envelope spectrum (ES) of the inner race fault rotates at 2100 rpm. (**a**) ES of the raw signal, (**b**) ES of the TQWT-processed signal (Q = 1.5, J = 10).

**Fig. 18:**
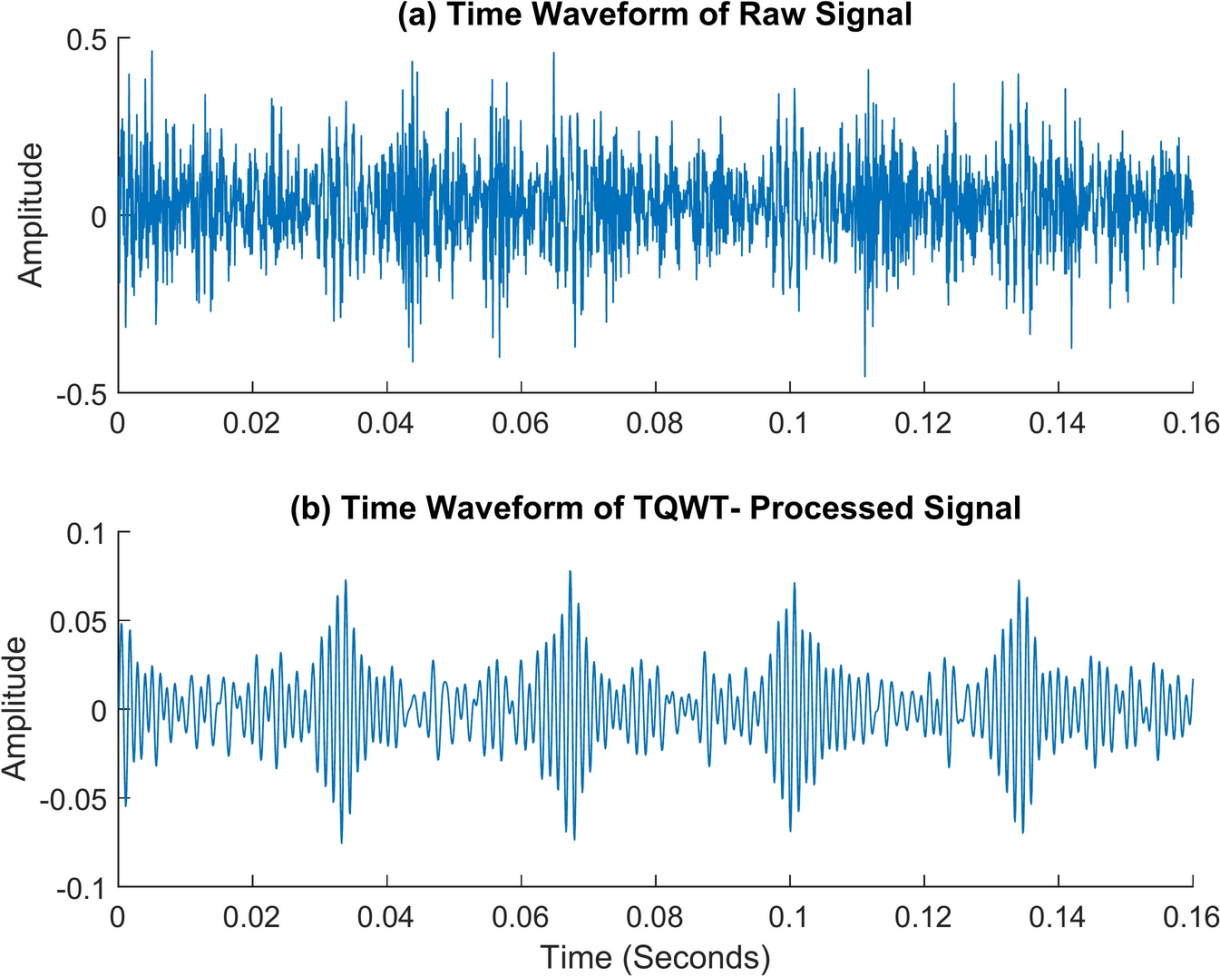
The time waveform (TW) of the inner race fault rotates at 2100 rpm. (**a**) TW of the raw signal (**b**) TW of the TQWT- processed signal (Q = 1.5, J = 10).

The following are Figs. 17 and 18. It demonstrates how to use TQWT to identify the inner race fault frequency. The TQWT parameters Q, R, and J are chosen to be 1.5, 3, and 10 respectively for that. The most appropriate detailed subband which contains the greatest energy is selected from among eleven subbands by TQWT, which decomposes the original signal into those. The inner race fault frequency is determined from the envelope spectrum of the chosen subband, as can be seen. The machine is rotating at 2100 rpm with no load when the vibration data utilized for this is acquired. Figure 17a shows the raw signal’s envelope spectrum. It is possible to see one or two frequency peaks, but they do not reveal much information about the fault frequency itself. Yet, if we look at the envelope spectrum of the TQWT-processed signal given in Fig. 17b, we can clearly identify the inner race fault frequency and associated harmonics. The inner race fault frequency of 346 Hz is found, and this value is really close to the theoretical value of 346.15 Hz determined using the formula given in Table 2. In the figure, *f*_*i*_ represents the inner race fault frequency, and *2f*_*i*_, *3f*_*i*_ represents its harmonic frequencies. The time waveform graph given in Fig. 18 illustrates how well TQWT has done in separating the necessary fault signal from a jumbled raw signal.

**Fig. 19:**
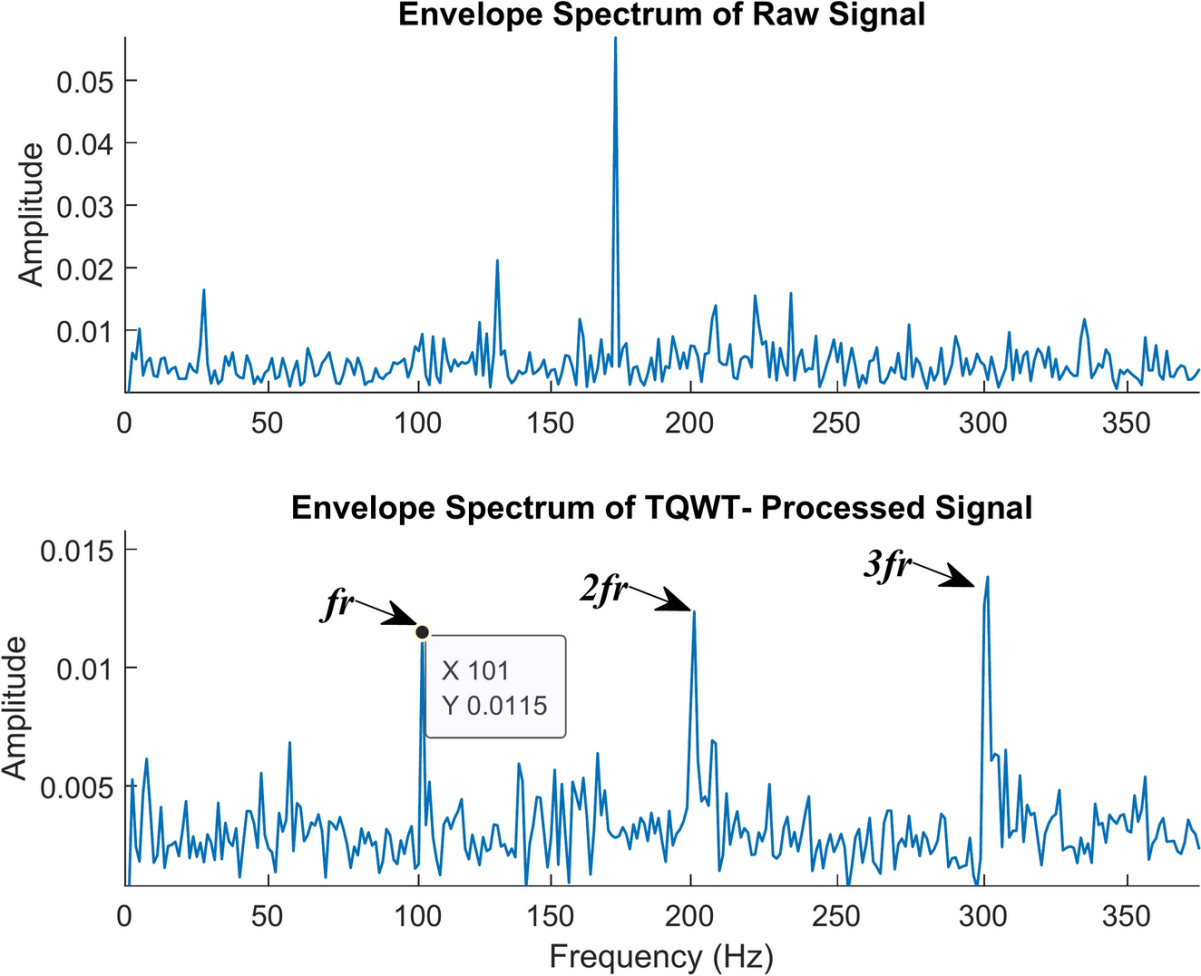
The envelope spectrum (ES) of the roller fault rotates at 2100 rpm. (**a**) ES of the raw signal, (**b**) ES of the TQWT-processed signal (Q = 1.5, J = 10).

**Fig. 20:**
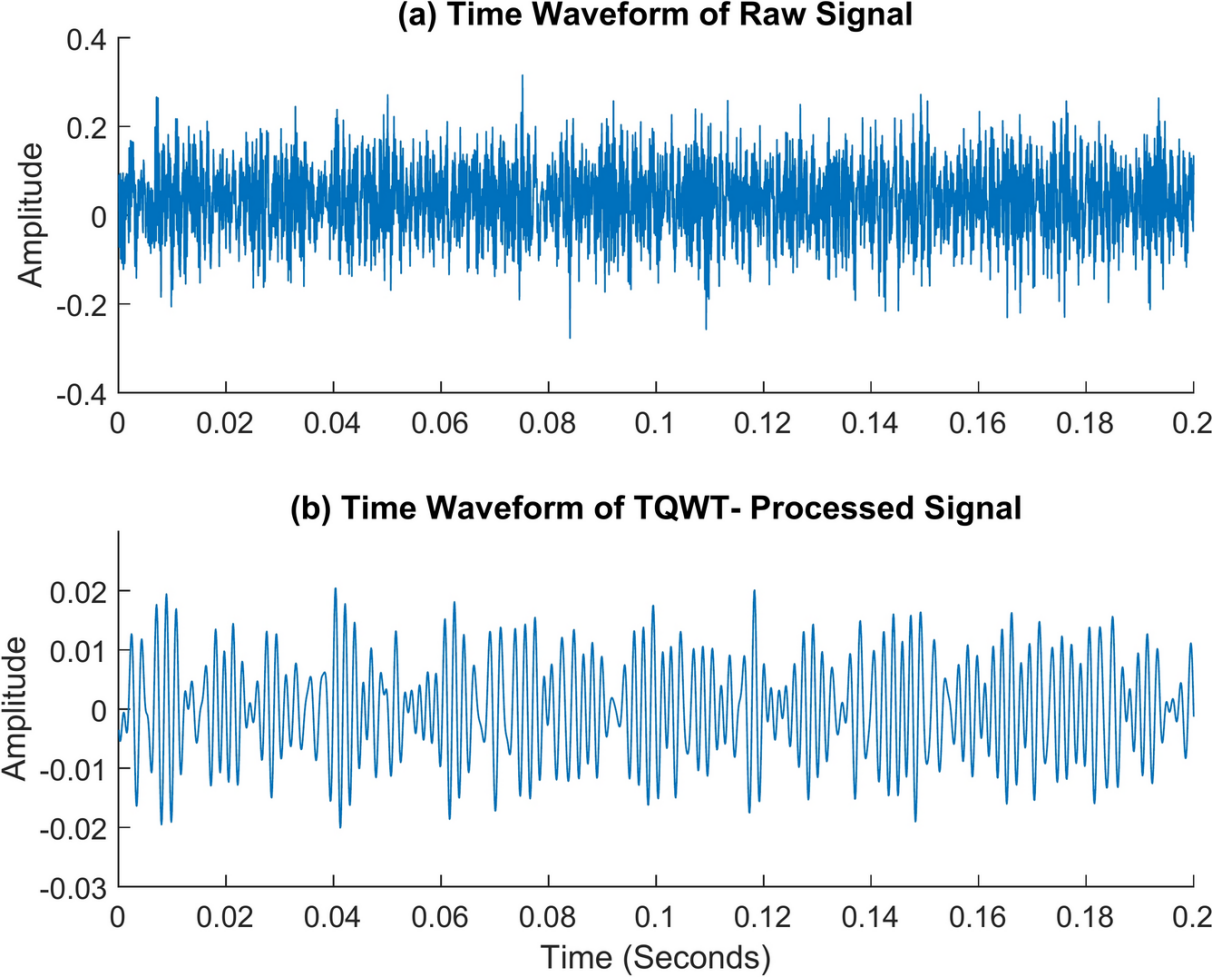
The time waveform (TW) of the roller fault rotates at 2100 rpm. (**a**) TW of the raw signal (**b**) TW of the TQWT- processed signal (Q = 1.5, J = 10).

The roller fault is the next and is shown in Figs. 19 and 20. It contrasts the envelope spectrum of a roller defect when the shaft is rotating at 2100 rpm and when there is no load. Figure 19a shows the envelope spectrum of the raw signal. There is only one frequency peak visible in this spectrum, and it has nothing to do with roller failure. The envelope spectrum of the TQWT-processed signal, with the parameters Q, R, and J being 1.5, 3, and 10 respectively, is shown in Fig. 19b. The roller fault frequency of 101 Hz is found, and this value is enormously close to the theoretical value of 100.45 Hz determined using the formula given in Table 2. The roller fault frequency and its harmonic frequency are quite easy to discern in that figure. When a roller fault frequency is represented by *f*_*r*_, its harmonic frequencies are represented by *2f*_*r*_ and *3f*_*r*_. Figure 20 shows the roller fault’s time waveform graph. Figure 20a shows the time waveform of the raw signal, and Fig. 20b shows the time waveform of the signal after TQWT processing.

**Fig. 21:**
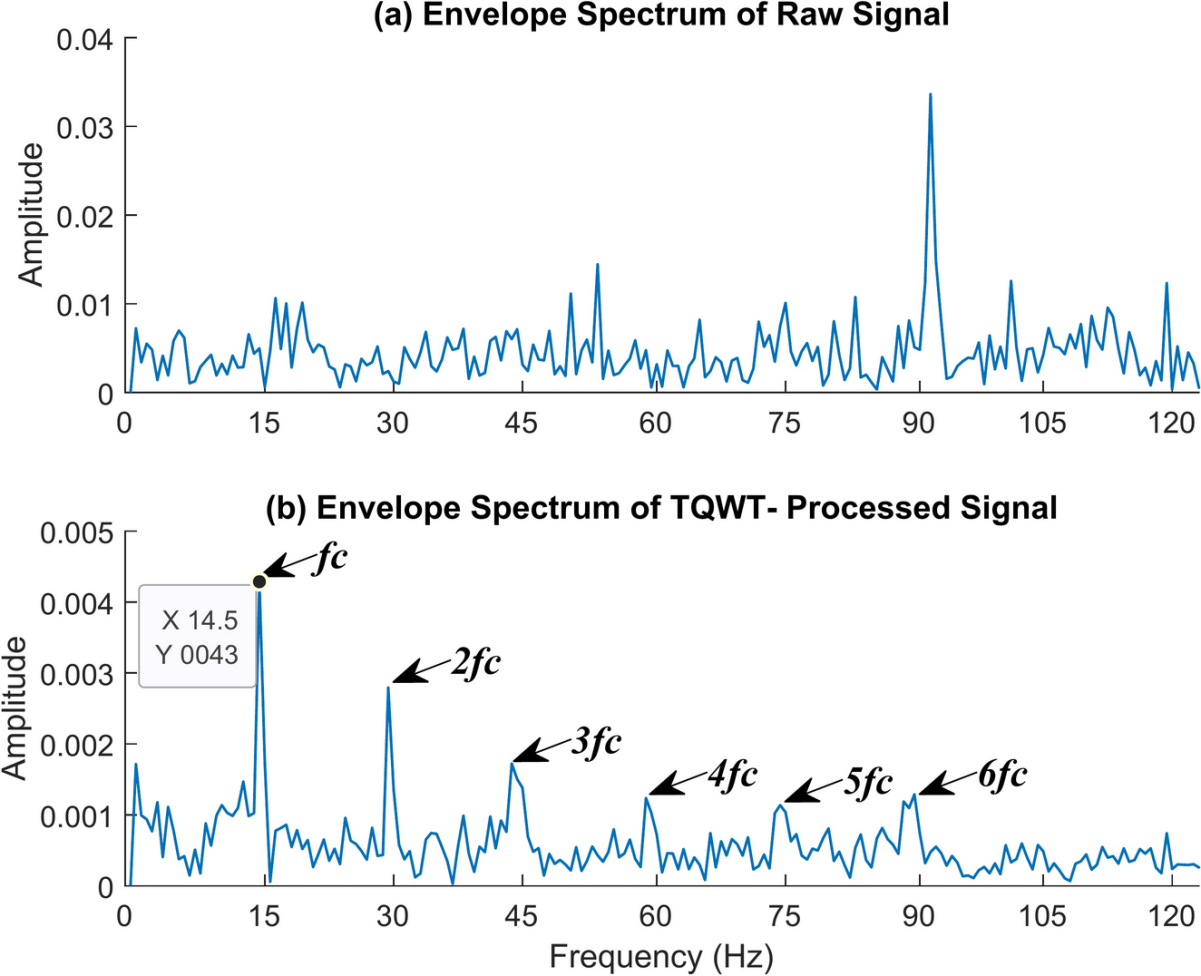
The envelope spectrum (ES) of the cage fault rotates at 2100 rpm. (**a**) ES of the raw signal, (**b**) ES of the TQWT-processed signal (Q = 1.5, J = 10).

**Fig. 22:**
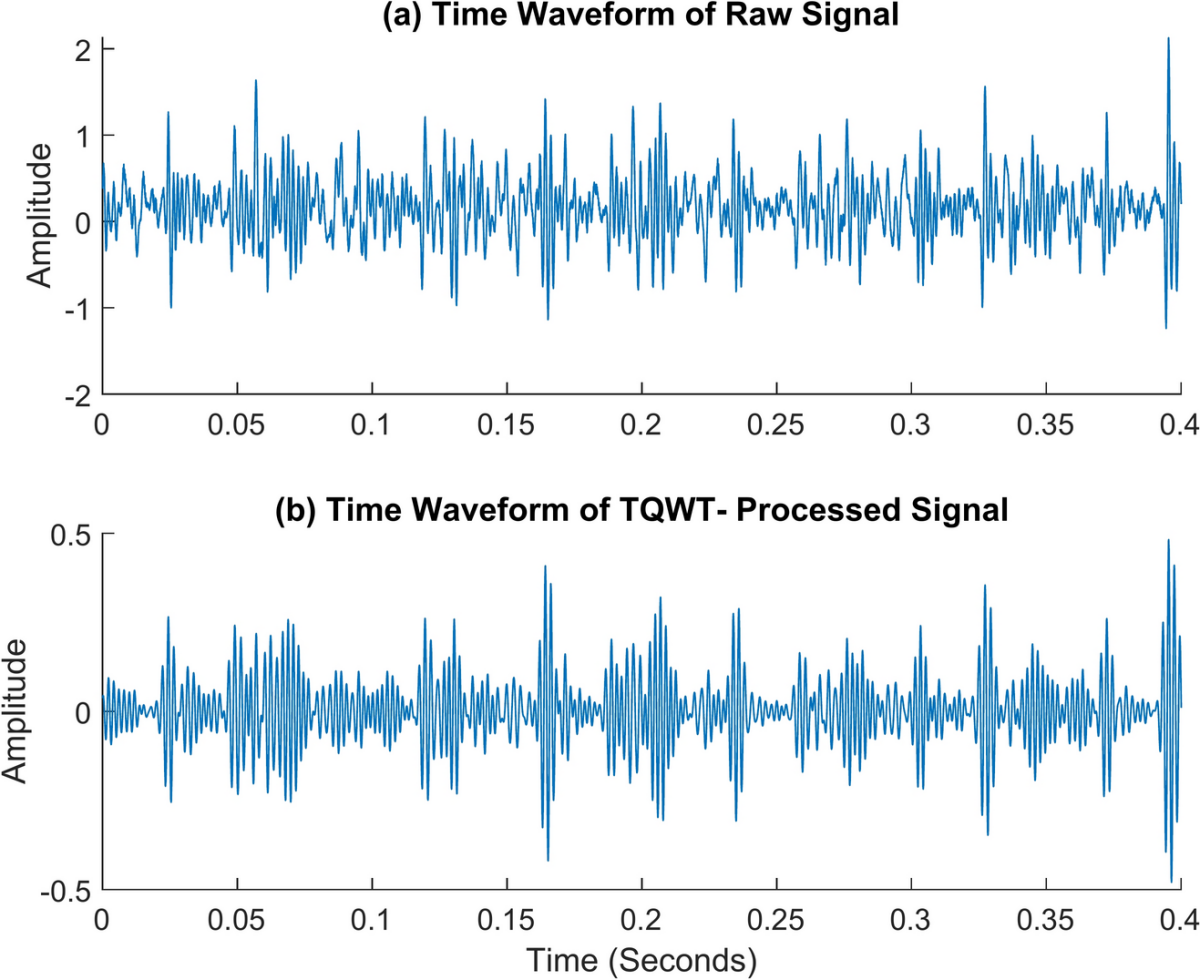
The time waveform (TW) of the cage fault rotates at 2100 rpm. (**a**) TW of the raw signal (**b**) TW of the TQWT- processed signal (Q = 1.5, J = 10).

The cage fault comes next. For that, the shaft speed of 2100 rpm and the no-load conditions are employed to capture the vibration data. The following TQWT parameters were used in this case: Q = 1.5, R = 3, and J = 10. The envelope spectrum of the raw signal and the TQWT-processed signal is given in Fig. 21. The envelope spectrum of the TQWT-processed signal accurately captured the cage fault frequency and the associated harmonic frequencies. The cage fault frequency of 14.5 Hz is found, and this value is remarkably close to the theoretical value of 14.63 Hz determined using the formula given in Table 2. In this case, *f*_*c*_ represents the cage fault frequency, and *2f*_*c*_*, 3f*_*c*_*, 4f*_*c*_*, 5f*_*c*_*,* and *6f*_*c*_ are its harmonic frequencies. The time waveform graph of the cage fault signal is given in Fig. 22.

Based on the experimental results shown in the aforementioned paragraphs, it can be inferred that the TQWT perfectly eliminates unwanted signals and noise, leaving just the fault signal to be recognized.

### Fault classification by LSTM

This is the second stage of the proposed method. In this stage, the LSTM approach for fault classification will be discussed. Here, five lakh data points gathered from bearings with various fault states are used to determine that. Out of the five lakh data points, the first one lakh corresponds to the normal bearing, followed by one lakh each for the inner ring fault, outer ring fault, roller fault, and cage fault. The LSTM network is implemented using Matlab R2022a. The data points for each fault condition are split into fifty batches with a window size of 2000 after being loaded into the Matlab live editor. The complete set of data must then be divided into training and testing groups. In this investigation, 80% of the datasets will be used for training and 20% for testing.

The LSTM network definition comes next. First, the suitable number of classes, hidden units, and input size is chosen for the experiment. The input size is 2000, there are 1000 hidden units, and there are 5 classes in this experiment. The network architecture is then created. In this experiment, five layers were used. The sequence input layer, LSTM layer, fully connected layer, softmax layer, and classification output layer are the ones mentioned before. In Table 3, each layer’s specifics are listed. The options for training are then presented. The solver for this experiment is designated as “adam,” the maximum number of epochs is set at 100, and the mini-batch size is set at 32. Then use these training choices to train the network. The LSTM network must be tested in the final phase. To do this, load the test dataset and categorize the various bearing defects.

**Table 3:** A 5 × 1 layer array with layers.

No	Layer	Specification
1	Sequence input	Sequence input with 2000 dimensions
2	LSTM	LSTM with 1000 hidden units
3	Fully connected	5 fully connected layers
4	Softmax	softmax
5	Classification output	crossentropyex

**Fig. 23:**
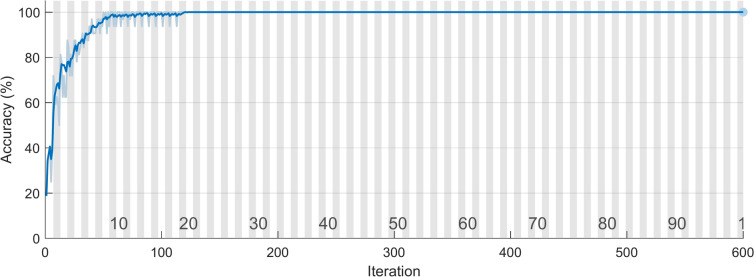
Training plot- accuracy vs iteration.

**Fig. 24:**
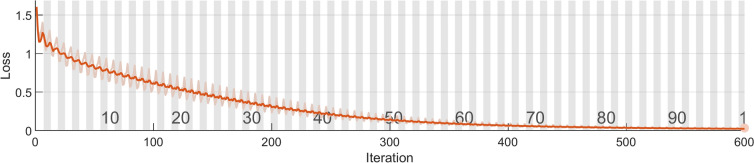
Training plot- loss vs iteration.

Now begin to categorize various faults present in the test bearing. Here, the signal processed using the TQWT approach serves as the input for the LSTM network. In Figs. 23 and 24, the training plots for the suggested approach are presented. Figure 23 plots accuracy against iteration, while Fig. 24 plots loss against iteration. It is clear from these figures that the network’s accuracy reached 100% at the 120th iteration, and the loss is completely eliminated after 500 iterations.

**Fig. 25:**
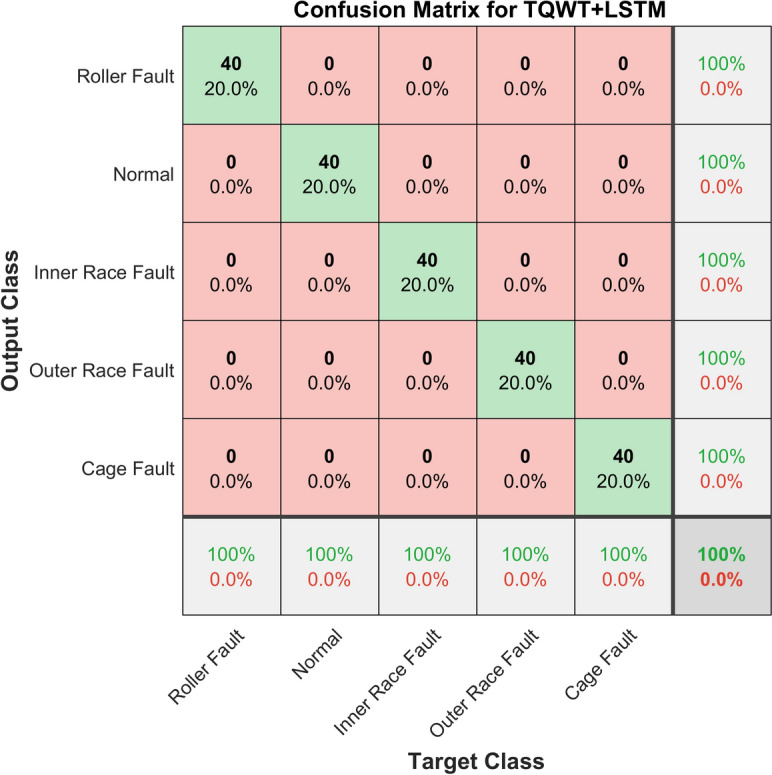
The Confusion Matrix of the experimental data using the proposed method (TQWT + LSTM).

The confusion matrix for the proposed method is shown in Fig. 25. A confusion matrix is a matrix that provides an overview of how well a machine learning model performs on a collection of test data. It is a way to show how many of the model’s predictions were correct and how many were not. The performance of classification models, which seek to predict a categorical label for every instance of an input, is frequently assessed using this method. It can be seen that, with 100% accuracy, the method proposed in this article perfectly diagnosed all the faults present in the taper roller bearing.

**Fig. 26:**
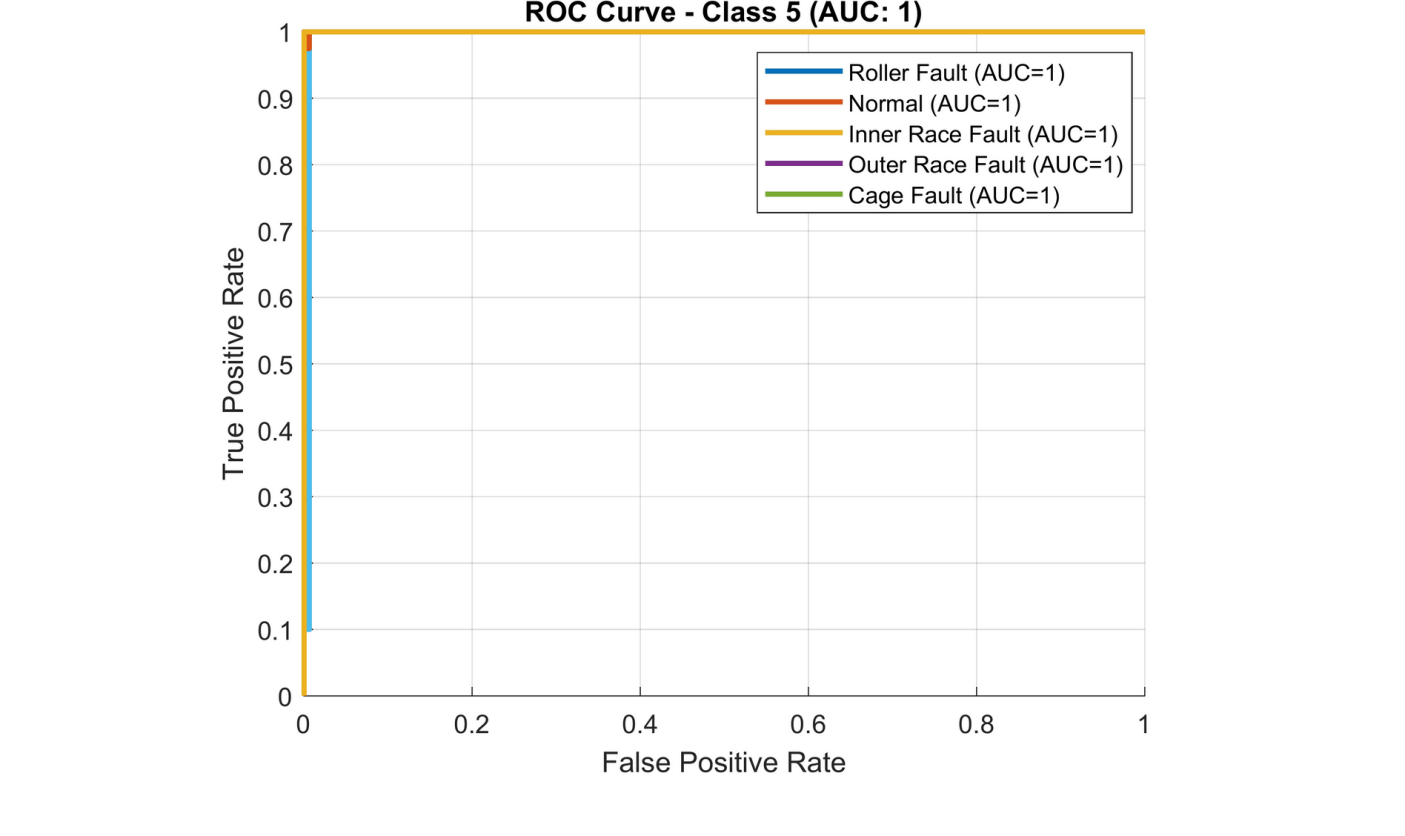
ROC curve of the experimental data using the proposed method (TQWT + LSTM).

The suggested method’s receiver operating characteristic curve (ROC) is depicted in Fig. 26. A ROC curve that is nearer the upper-left corner indicates that the model is performing better. With a range of 0 to 1, the area under the curve (AUC) measures this performance. Random guessing is indicated by an AUC of 0.5, whereas perfect categorization is indicated by an AUC of 1. It is clear that the proposed classifier is fully able to distinguish between all positive and negative class points because the area under the curve (AUC) is one.

Besides accuracy, there are other matrices that are crucial for a successful model evaluation. The efficiency of the constructed model is also assessed using precision rate, recall rate or sensitivity, specificity, and F1 score. The precision rate is the ratio of correctly predicted positive observations to all positively predicted observations. The recall rate or sensitivity is the proportion of correctly predicted positive observations to all observations in the actual class. The ratio of correctly identified negative data to actual negative data is known as the classifier’s specificity, while the F1 score is the harmonic average of the precision rate and the recall rate. Equations 18–21 provide the mathematical expressions for these matrices. The outcomes of the aforementioned matrices in this experiment are displayed in section “Comparison study”.

Precision rate,

18 $$P = \frac{True\;Positive}{{\left( {True\;Positive + False\;Positive} \right)}}$$

True Positive Rate/ Sensitivity /Recall,

19 $$R = \frac{True\;Positive}{{\left( {True\;Positive + False\;Negative} \right)}}$$

True Negative Rate/ Specificity,

20 $$S = \frac{True\;Negative}{{\left( {True\;Negative + False\;Positive} \right)}}$$

F1 Score,

21 $$F1 = 2*\frac{{\left( {Precision*Recall} \right)}}{{\left( {Precision + Recall} \right)}}$$

### Comparison study

**Fig. 27:**
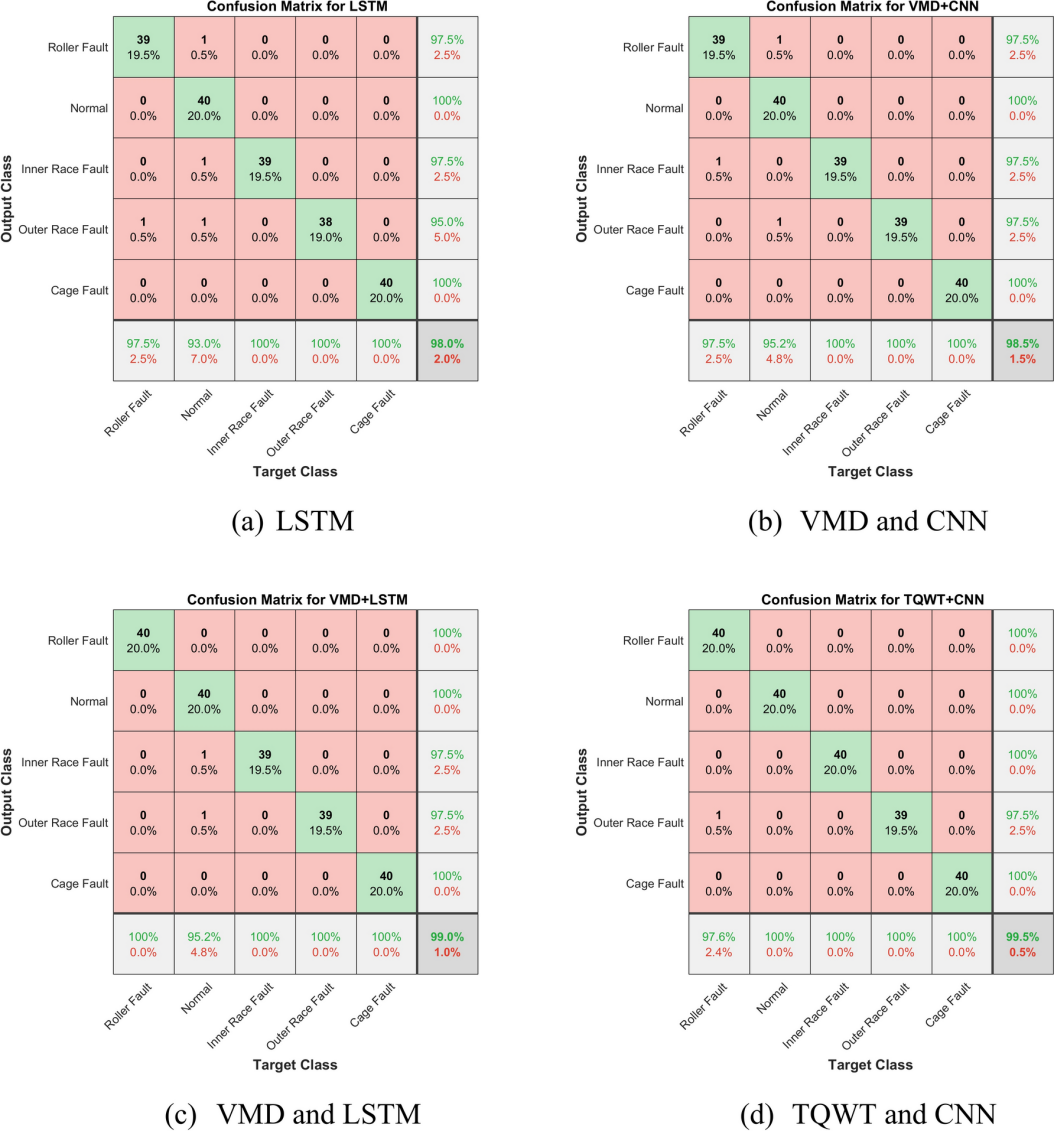
Confusion matrix of experimental data using different frameworks.

**Fig. 28:**
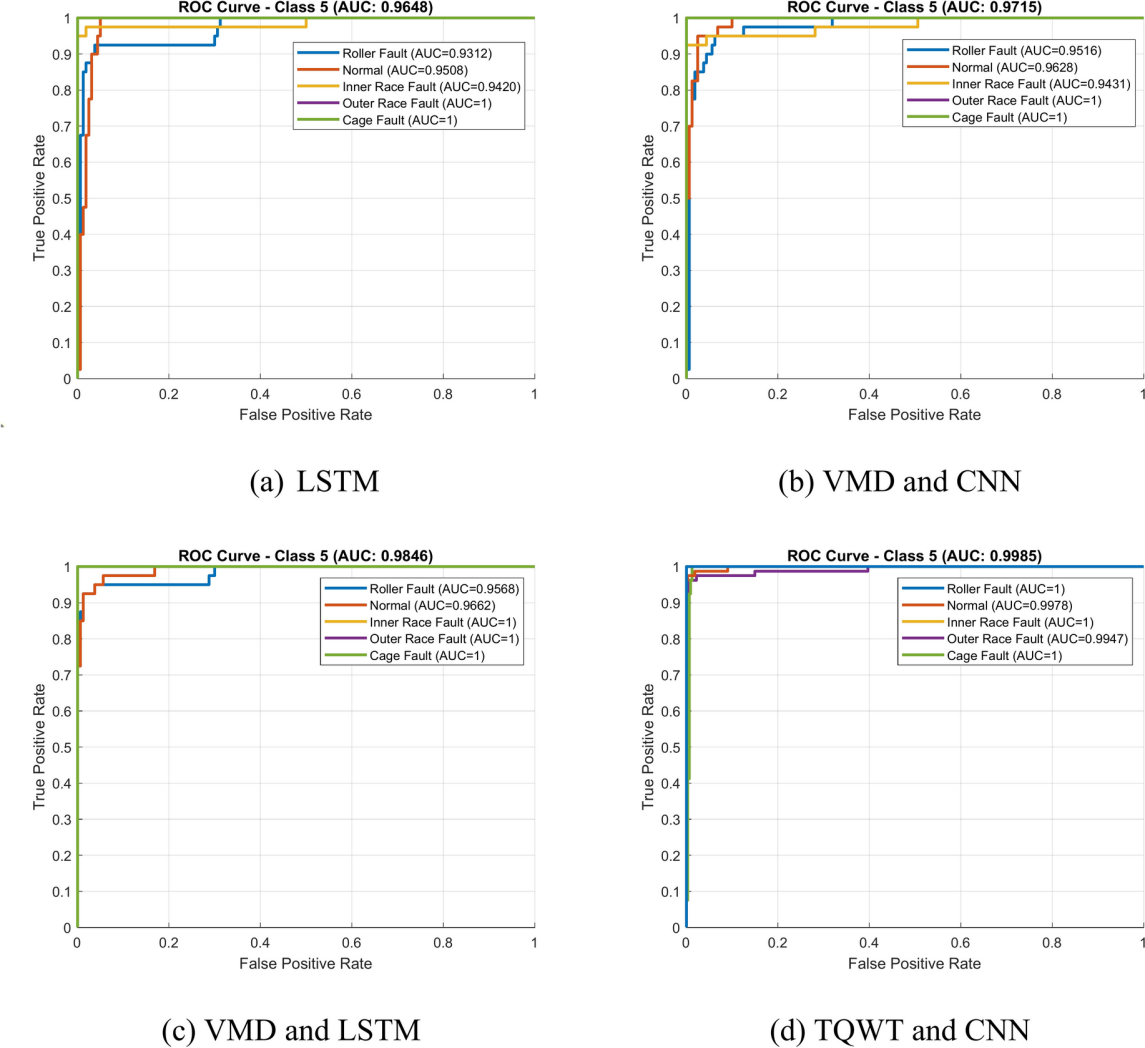
ROC curve of experimental data using different frameworks.

An explanation of any methodology is incomplete without a clear comparison study. So, this section provides a comparative study of the proposed methodology with four other well-known methods. First, the accuracy of fault classification using just the LSTM network is discussed. Here, there is no signal processing applied to the input for the LSTM; it is simply the raw vibration signal collected from the experimental setup. It can be seen that LSTM perfectly classified the various defects, including normal (healthy bearing), inner ring fault, outer ring fault, roller fault, and cage fault. Figure 27a provides the confusion matrix for the LSTM network. The figure shows that 40 batches of normal bearing, and 40 batches of cage fault are all accurately classified with zero error. However, a batch of roller faults and a batch of inner race faults were misclassified as the normal bearing, and two batches of outer race faults were misclassified as a roller fault and a normal bearing. Moreover, the network accuracy is limited to 98%. It is evident that the categorization accuracy decreases without TQWT. The ROC curve for experimental data with the LSTM network is shown in Fig. 28a. The average AUC across all five classes, as can be seen, is 0.9648, which is smaller than the suggested method’s AUC.

Secondly, the classification accuracy of the suggested method is compared with the classification accuracy of another sophisticated combination. This combination is made using the variational mode decomposition (VMD) approach and Convolutional Neural Network (CNN) architecture. A detailed theory of the VMD method and CNN architecture is given in reference^43,44^. In this case, the CNN network uses the signal that was processed using the VMD technique as its input data. The confusion matrix for the taper roller bearing fault classification using the combined application of VMD and CNN techniques is shown in Fig. 27b. As can be observed, one batch of roller fault and one batch of outer race fault were misclassified as the normal bearing, and one batch of inner race fault was misclassified as the roller fault. Moreover, the network accuracy is limited to 98.5%. This is a lower percentage of classification accuracy than that obtained with the recommended approach. The experimental data’s ROC curve employing VMD and CNN methodology is shown in Fig. 28b. As can be observed, the average AUC across all five classes is 0.9715, which is lower than the suggested method’s AUC.

Next, the classification accuracy of the suggested strategy is contrasted with the classification accuracy of another advanced combination. This combination is built using the VMD approach and the LSTM framework. Here, the signal processed using the VMD approach serves as the input data for the LSTM network. The confusion matrix for the taper roller bearing fault classification using the combined application of VMD and LSTM techniques is shown in Fig. 27c. It can be seen that one batch of inner race fault and one batch of outer race fault were misclassified as the normal bearing, and the network accuracy is limited to 99%. This percentage of categorization accuracy is lower than what the suggested method achieves. The experimental data’s ROC curve employing the combination of VMD and LSTM is shown in Fig. 28c. As can be observed, the average AUC across all five classes is 0.9846, which is lower than the suggested method’s AUC.

Finally, the suggested strategy’s classification accuracy is then compared to another combination’s classification accuracy. The TQWT method and CNN framework are used to create this combo. Figure 27d displays the confusion matrix for the taper roller bearing fault classification with the combined use of TQWT and CNN approaches. As can be observed, one batch of outer race faults was misclassified as the roller fault, and the maximum network accuracy obtained is 99.5%. This categorization accuracy percentage is less than that of the recommended approach. The experimental data’s ROC curve is displayed in Fig. 28d. As can be seen, the average AUC across all five classes is 0.9985, which is slightly lower than the recommended method’s AUC.

**Fig. 29:**
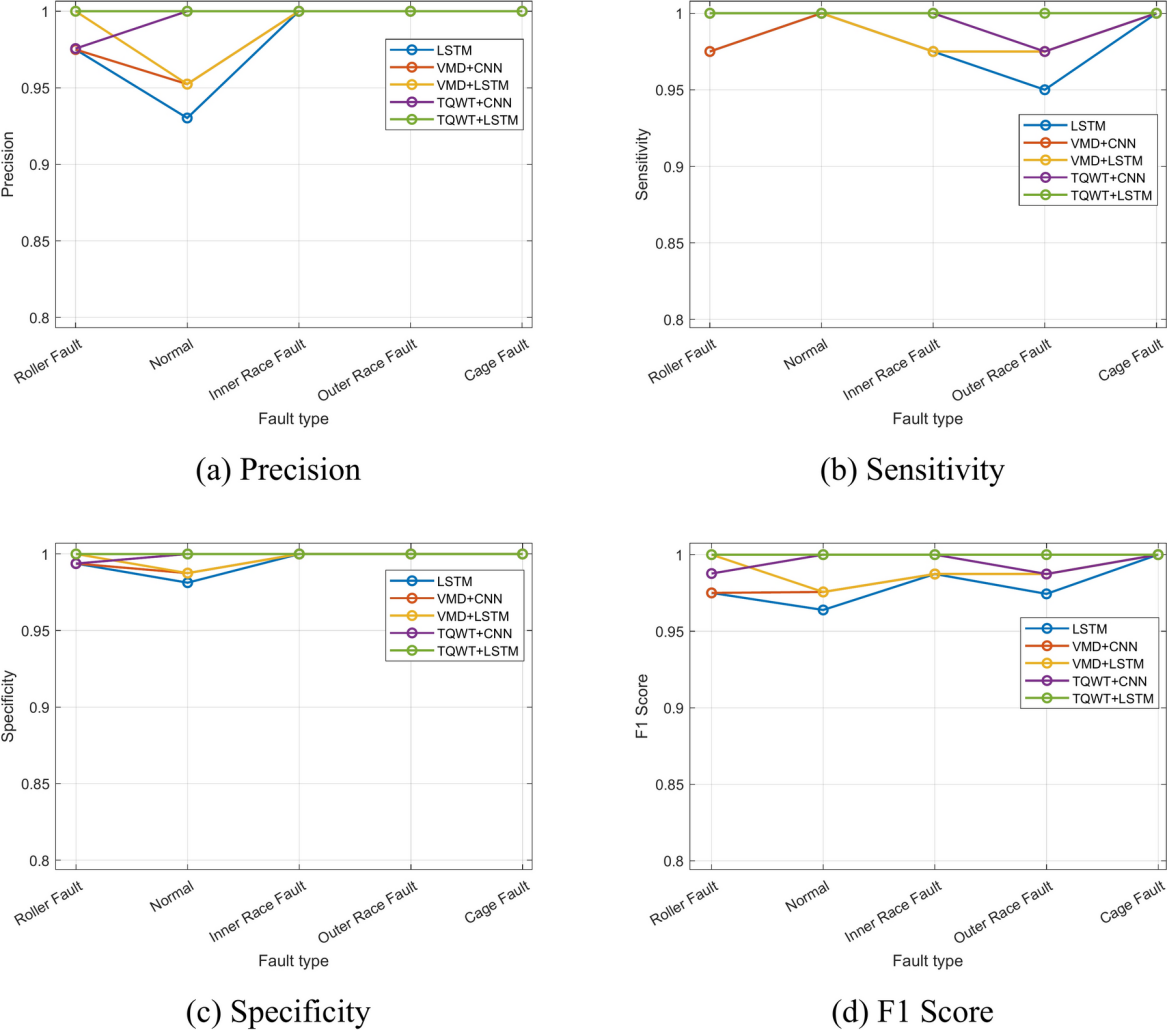
Precision, sensitivity, specificity, and F1 score of various fault conditions using different frameworks.

**Fig. 30:**
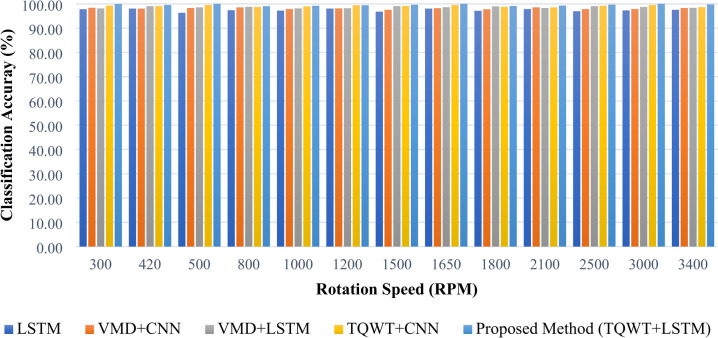
Classification accuracy of the experimental data at different rotating speeds.

Using five distinct combinations of approaches, Fig. 29 and Table 4 compare the precision, recall or sensitivity, specificity, and F1 score of various fault circumstances. Additionally, Table 4 shows the computational efficiency of the proposed method. It is evident that the suggested technique outperforms every matrix. Figure 30 and Table 5 both provide an overview of the categorization accuracies of the proposed methodology at different operating conditions. As a result, readers may quickly compare the suggested approach’s categorization accuracy to other comparable methods at various rotation speeds.

**Table 4:** Model evaluation using precision, sensitivity, specificity, F1 score, train and test time.

Methods	Precision %	Sensitivity %	Specificity %	F1 score %	Train time (s)	Test time (s)
LSTM	98.10	98.00	99.50	98.01	272	0.986
VMD + CNN	98.55	98.50	99.63	98.51	155	0.865
VMD + LSTM	99.05	99.00	99.75	99.01	218	0.913
TQWT + CNN	99.51	99.50	99.88	99.50	105	0.312
TQWT + LSTM	100	100	100	100	107	0.354

**Table 5:** Classification accuracy of the experimental data at different rotating speeds.

Trial No	Rotation speed (RPM)	LSTM (%)	VMD + CNN (%)	VMD + LSTM (%)	TQWT + CNN (%)	Proposed method TQWT + LSTM (%)
1	300	97.80	98.40	98.10	99.30	99.90
2	420	98.00	98.00	99.00	99.00	99.50
3	500	96.30	98.30	98.50	99.50	100.00
4	800	97.40	98.50	98.80	98.70	99.00
5	1000	97.20	97.90	98.10	98.90	99.20
6	1200	98.00	98.10	98.20	99.40	99.40
7	1500	96.80	97.50	99.00	99.10	99.60
8	1650	98.00	98.20	98.60	99.50	100.00
9	1800	97.10	97.80	98.90	98.80	99.10
10	2100	97.90	98.50	98.30	98.50	99.30
11	2500	97.00	97.90	99.00	99.20	99.70
12	3000	97.30	97.90	98.70	99.50	100.00
13	3400	97.50	98.30	98.40	98.60	99.80

**Fig. 31:**
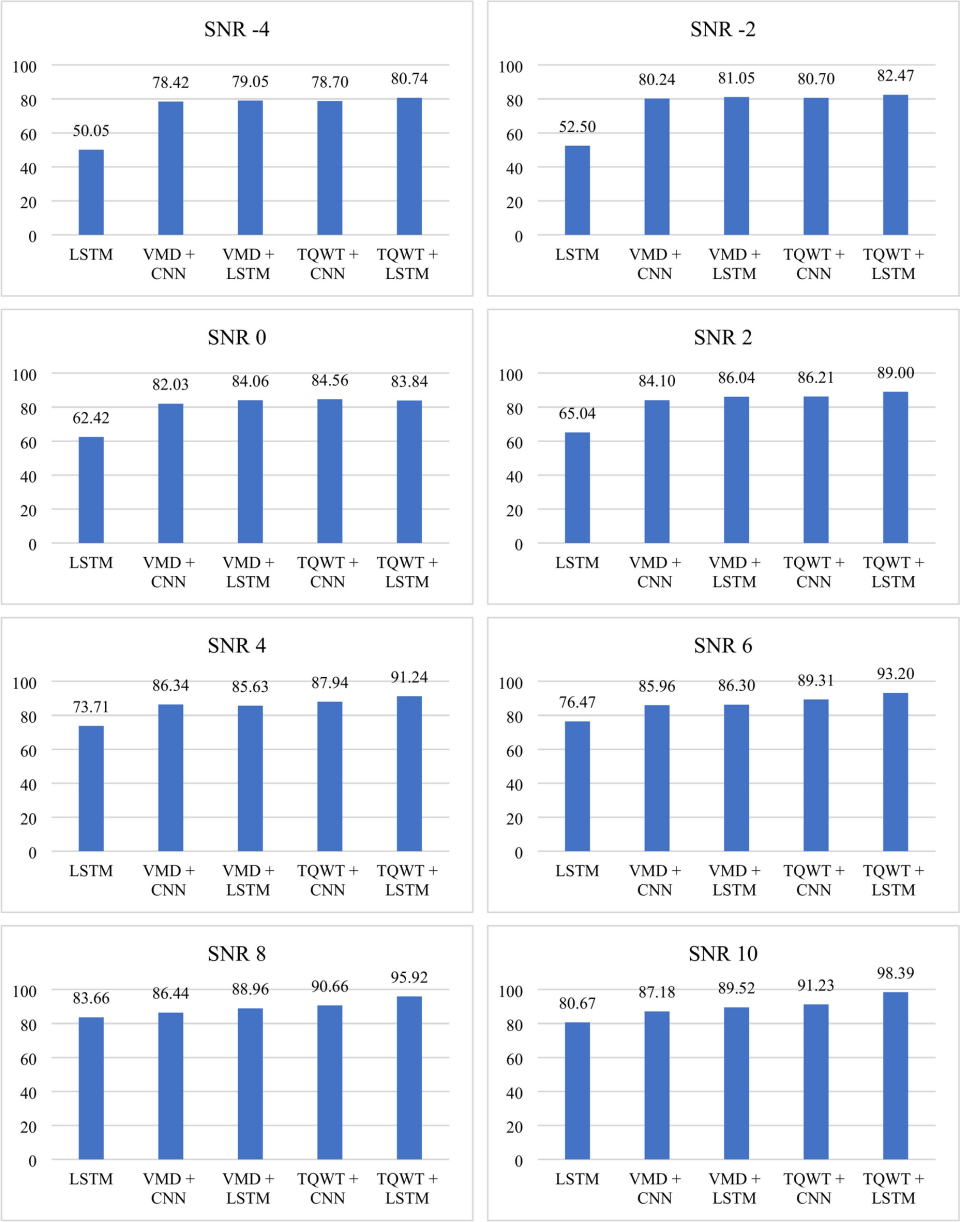
Accuracy percentage of the experimental data under various levels of noise.

It would be advantageous to evaluate the model’s resilience to uncertainty in the real world. For that, the classification accuracy of the proposed technique at various noise levels is also evaluated. This was accomplished by adding Gaussian white noise to the original vibration signals with varying signal-to-noise ratios (SNRs) between − 4 and 10 dB. The expression for SNR is given in Eq. 22. Where *P*_*s*_ represents the signal power and *P*_*n*_ represents the noise power. It is quite challenging to extract valuable features from the composite noise signal when SNR is less than zero, because the energy of the noise signal is higher than the original signal. The average accuracy of the five approaches in various noise situations is displayed in Fig. 31. It is evident that as the SNR increases, so does the accuracy. However, the accuracy of the suggested model increases to 98.39% when SNR = 10, which is significantly greater than that of other intelligent diagnosis models.

Signal-to-noise ratio,

22 $$SNR = 10 log_{10} \frac{{P_{s} }}{{P_{n} }}$$

These comparisons given in this section makes it clear that the combination of TQWT and LSTM is a technique that promotes the development of the ideal fault classification method in the field, which is useful not only for diagnosing taper roller bearing faults but also for diagnosing faults of different kinds and different sizes of rolling element bearings, including ball bearings, cylindrical roller bearings, and spherical roller bearings. Since the methodology for fault diagnosis and fault classification described in this work is straightforward and accurate, it may also be used to identify and categorize defects in rotating parts of different sizes, including shafts, gears, and rotors.

## Feasibility for real-time applications

This section explains the feasibility of implementing the proposed fault diagnostic method in modern industry with the help of the Industrial Internet of Things (IIoT) for online condition monitoring in real-time applications. IIoT-based condition monitoring systems gather data from the machinery in real time, analyze it, and evaluate it in accordance with the recommended performance levels. If the machine’s performance level does not meet the predetermined limits, the platform promptly notifies the maintenance manager and conveys the problem with the findings of the fault classifier.

**Fig. 32:**
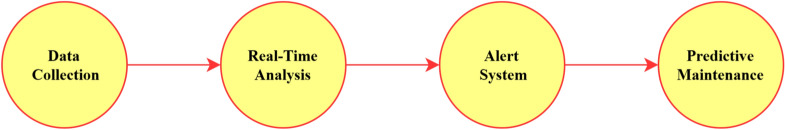
Online condition monitoring for industrial application.

There are four processes involved in online condition monitoring, as illustrated in Fig. 32. They are data collection, real-time analysis, alert system, and predictive maintenance. Sensors are placed strategically on equipment to evaluate key performance characteristics. These sensors gather data continuously and transmit it to a central monitoring system. Real-time analysis of the collected data is done with the proposed method. This study facilitates the identification of patterns and anomalies that may indicate potential problems. Every time it detects an anomaly or deviations from the norm, the system notifies or issues alerts. Maintenance teams can address issues before they become costly malfunctions by employing these alerts. Online Condition Monitoring schedules maintenance actions shortly before a failure is likely to occur by using predictive analytics. This approach reduces unplanned downtime and maximizes maintenance resources.

Finding anomalous circumstances or weaknesses in machinery or systems is referred to as fault detection. The main objective is to identify deviations from regular operation as soon as possible in order to avoid significant breakdowns or inefficiencies. Finding the underlying cause of an issue that has been recognized is the goal of fault diagnostics. After a fault is identified, diagnostics seeks to identify the precise cause and kind of the problem so that focused remedial measures can be taken. This research contributes an effective defect detection and diagnostics which leads to increased safety, cost savings, improved equipment reliability, and operational efficiency.

The potential challenges in integrating the fault diagnostics system into an operational plant include data overload- managing large amounts of data from sensors and monitoring systems, integration problems of the system with existing infrastructure, the possibility for false alarms, and the skill requirements to make informed decisions.

## Conclusions

In this work, a novel approach to the diagnosis and categorization of taper roller bearing faults is suggested. The results of the simulation study and experimental analysis are summarized in the following sentences. A framework that correctly handles the signal collected from the equipment is necessary for an advanced fault diagnosis approach. TQWT, a signal processing framework, does this section pretty well. The TQWT can decompose the signal without sacrificing any of the properties necessary for fault identification, and it can very precisely remove all the undesired components and noise of a mixed-up signal. Similarly, the literature review described in Section “Introduction” can be used to understand the increasing potential of deep learning in fault classification procedures. This work uses the extremely sophisticated deep learning technique LSTM network to categorize taper roller bearing faults.

Based on the outcomes of this experiment, it has been determined that the TQWT and LSTM combination is 100% accurate at classifying various types of bearing problems. Similar to this, a comparison study with the outcomes of the other four extremely sophisticated and well-known methods is provided in Section “Comparison study” to show the superiority of the suggested methodology. It is evident from all of this that the method put forward in this paper is a tremendous success and can be utilized for the diagnosis and categorization of faults in taper roller bearings as well as many other moving parts including gears, shafts, and other similar components.

## Data Availability

The datasets generated and/or analysed during the current study are not publicly available due to institution norms but are available with the corresponding author on reasonable request.
